# Alchemy in Nature: The Role of *Lawsonia inermis* Extract Choice in Crafting Potent Anticancer Metal Nanoparticles

**DOI:** 10.1021/acsami.4c19585

**Published:** 2025-01-11

**Authors:** Rana Ahmed El-Fitiany, Riham El Nahas, Seba Al Balkhi, Shouq Aljaeedi, Afra Alblooshi, Fathy M. Hassan, Abbas Khaleel, Abdelouahid Samadi, Mohammad A. Khasawneh

**Affiliations:** † Department of Chemistry, College of Science, United Arab Emirates University, Al Ain, 15551, United Arab Emirates; ‡ Pharmacognosy Department, Faculty of Pharmacy, Egyptian Chinese University, Cairo, 19346, Egypt

**Keywords:** Lawsonia inermis, antioxidant, skin cancer, nanoparticles, phytochemical profiling, LC/MS/MS

## Abstract

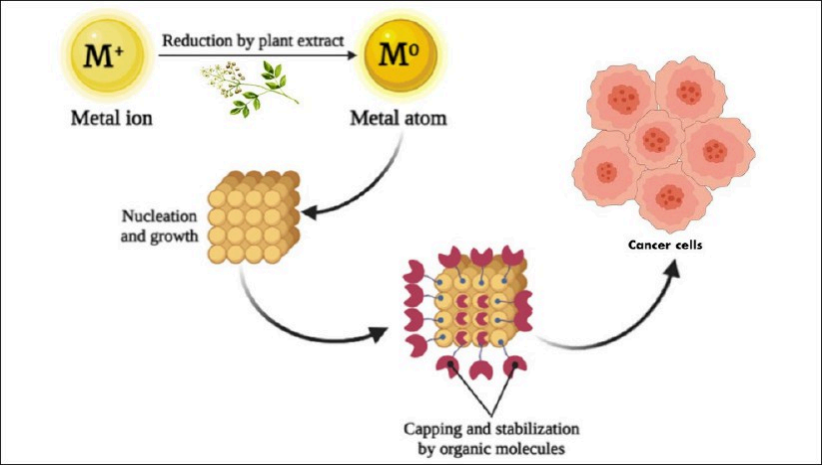

Phyto-nanotechnology provides an eco-friendly approach for synthesizing biocompatible metal nanoparticles (NPs) with therapeutic potential. *Lawsonia inermis* (LI) has been historically valued for its diverse medicinal applications, especially its exceptional biological potency against various skin diseases, attributed to its rich abundance of bioactive compounds. Therefore, herein, plant-based iron and zinc NPs were biofabricated via sustainable and simple methods, using crude extracts of the aerial parts of LI as reducing, coating, and stabilizing agents. Since the extraction method affects the type of extracted phytocompounds, two extraction approaches—aqueous and hydro-alcoholic—were applied to determine the influence of the extraction route on the physicochemical and biological properties of the formed NPs. These properties were characterized via various analytical techniques and assays. The UV–Vis spectra revealed absorption bands ranging from 265 to 270 nm, while FT-IR confirmed the successful coating of the NPs with the extracts’ phytochemicals, validating the biofabrication of the proposed NPs. The alcoholic-based NPs displayed higher total phenolic content, total flavonoid content, and antioxidant effect compared to their aqueous-based counterparts, reaching up to 55.13 μg of GAE/1 mg of dry weight (DW), 30.48 μg of QU/1 mg of DW, and IC_50_ of 46.02 μg/mL, respectively. All tested samples, except for Fe NPs, displayed significant cytotoxic effects against skin cancer, resulting in a cell viability as low as 1% at 1000 μg/mL. QTOF-LC/MS/MS analyses of LI extracts revealed tentative identification of more than 100 metabolites with phenolic compounds representing the largest share. Orthogonal Projections to Latent Structures Discriminant Analysis modeling revealed a clear separation between both extracts, with more than 40 marker compounds. The results indicated that both extracts were effective for the green synthesis of Fe and Zn NPs for biomedical applications, with the alcoholic extract of LI as a superior coating candidate and the aqueous extract as a stronger reducing agent. This work showcases the influence of extraction protocols on physicochemical and biological characteristics of the resulting nanoparticles.

## 1 Introduction

The era of nanotechnology has incorporated nanoparticles (NPs) in countless applications in essentially all modern technological fields, including medicine, agriculture, the environment, energy, and space. Since the past few years, the synthesis of eco-friendly, biologically active, and relatively safe NPs based on natural resources, such as plants or microorganisms, has emerged in medical applications. Several studies have shown promising findings of the natural-based NPs in terms of enhanced particle size, uniformity, purity, stability, biocompatibility, safety, and biological effects, in addition to being cost-effective over the chemically synthesized ones. These unique characters are assumed to be due to the richness of these natural materials with various biomolecules that act as reducing, capping, and stabilizing agents for the NPs.^1^

Plant extracts have shown several advantages over microorganisms in the green synthesis process. These extracts offer a faster rate of synthesis, higher suitability for upscaling, and better biocompatibility to the human body. The chemical constituents of these plants are the driving force for the synthesis of phytochemical-based NPs because they contain glycosides, carbohydrates, polyphenols, alkaloids, terpenes, flavonoids, phenolic acids, coenzymes, and proteins. These phytochemicals offer high diversity of functional groups, such as aldehydes, ketones, hydroxyl, carbonyl, carboxyl, and amino groups, which have crucial functions on the surface of the biofabricated NPs. These functions include reduction of metal precursors and inhibition of the aggregation of the formed NPs to maintain their stabilization, and finally they are excellent linkers and binders for many biological elements, like RNAs, DNAs, proteins, antibodies, antigens, enzymes, and polymers, making them excellent candidates for biomedical applications.^1,2^ In fact, the tiny sizes of the plant-based NPs enhance their capability to penetrate the smallest blood vessels, capillaries, barriers, and junctions, such as the blood brain barrier (BBB), to reach the central nervous system. This leads to improvement and enhancement of the solubility, bioavailability, distribution, and stability of these plant-based therapeutic NPs in the body, which makes them exceptional candidates for drug development and delivery research studies. Consequently, numerous research works have been emerging to design plant- or phytochemical-mediated NPs to enhance their biological bioavailability and effectiveness. Many studies have shown that plant-based NPs demonstrate more promising pharmacological activities than conventional plant crude extracts. These activities include antibacterial, antiviral, antifungal, and anticancer capacities, in addition to other diseases such as the neurodegenerative disorders.^3−7^

The physicochemical characters and yields of the biosynthesized NPs are affected by the synthesis environment including rate of synthesis, nature of the plant extract (alcoholic or aqueous), concentration, pH, temperature, and type of metal (and its concentration).^1,8,9^ Furthermore, the method of synthesis itself has a great influence since there are three different techniques for the biosynthesis of NPs using plants. The first one is the intracellular method, which is accomplished inside the tissues of the plants by adding the plant in an environment that is rich with the metal of interest. As the metal particles accumulate in the cells of the plants, they will be converted to NPs through their phytochemical content. In contrast, the second method is achieved extracellularly by utilizing extracts of plants or their specific organs. Finally, the third technique involves specific phytochemicals that are isolated from plants in the fabrication process. The second approach is the most common technique due to its ease of processing and tendency to scale-up since it is done by adding the plant extract to the solution of metal precursor at suitable pH and temperature.^9−11^ The process of synthesis itself consists of three phases; the first step is the reduction of the metal precursor to form metal atoms. The second stage is the nucleation process, which is started by the formed atoms. The third stage is the growth of the particles through sticking of the small particles on the larger ones, then the process is ended to obtain the final NPs.^9,12^ The synthesized NPs should be exposed to characterization via different techniques to ensure their quality (size, shape, crystallinity, surface coating, and surface charge). These techniques include Fourier Transform Infrared (FTIR) spectroscopy, Ultra-Violet Visible (UV–vis) spectroscopy, Scanning electron microscopy (SEM), X-ray diffraction (XRD), Transmission electron microscopy (TEM), Atomic Force Microscopy (AFM), and Dynamic light scattering (DLS).^7^

Several metals and their oxides are used in the synthesis of plant-based NPs; the most common ones are silver, gold, zinc, copper, iron, nickel, palladium, platinum, and selenium. In this project, iron and zinc metals were utilized for the synthesis of green NPs. In fact, iron, zinc, and their oxide NPs are considered to be among the interesting and promising metals in the fabrication of natural-based NPs, which demonstrates unique characteristics related to various applications of these NPs in biomedicine, bioremediation, cosmetics, and materials engineering. For example, zinc and iron plant-based fabricated NPs presented significant pharmacological activities including antioxidant and anticancer activities.^13−17^ Very recent studies revealed that zinc and iron NPs, mediated by different extracts of *Calotropis procera* and *Leptadenia pyrotechnica* aerial parts, exhibited significant antioxidant and skin anticancer effects, reaching radical scavenging effects and cell viabilities up to 99% at 100 μg/mL and 0.61% at 1000 μg/mL, respectively.^18,19^

*Lawsonia inermis* (LI; henna) was used in this project as a reducing, capping, and stabilizing agent. This plant is a species of the family Lythraceae. It is used traditionally as a natural dye for hair, skin, and fingernails. In addition, it is used in folk medicine in several countries of Asia and Africa for the treatment of several diseases, such as skin diseases, chickenpox, smallpox, tumors, and bacterial infections. Many research studies confirmed most of these medicinal uses by showing that the plant demonstrated several pharmacological effects, such as antibacterial, antifungal, anti-inflammatory, antioxidant, and cytotoxic activities. Also, it has shown superior biological effects against several skin disorders including cancer compared to other plant species. These activities are assumed to be related to the chemical compounds found in the plant, belonging to vast chemical classes as phenolics, alkaloids, proteins, fatty acids, triterpenoids, and sterols.^20^

Cancer is one of the major health concerns worldwide, and therefore the search for new anticancer agents with high safety profiles is necessary nowadays, especially that it is suggested that by 2050 there will be a 50% increase in the cancer cases according to the data acquired from the National Cancer Institute.^21^ As mentioned above, green NPs, in general, can penetrate and travel through the cells of the human body due to their tiny size, leading to enhanced intracellular and intranuclear bioavailability. In addition, iron and zinc NPs have shown significant anticancer effects, and LI extracts also have shown promising anticancer and skin treatment activity.^13−16,18−20^ Therefore, we propose that this combination could result in the development of effective, natural-based NPs for skin anticancer applications.

Because it is well-known that different extraction solvents extract distinct active constituents from plants, we assume that the extraction protocol will affect the physicochemical and biological characteristics of their corresponding green NPs. In fact, we did not find any study that addressed this issue or even resonated why they followed a specific extraction protocol rather than others. We believe it is important to investigate different extract types to get the most possible active green NPs for our aimed application.^22^ Furthermore, LI was selected in this study due to its well-known potency against skin diseases, especially its aerial parts.^20^ Therefore, the aim of this project is to carry out biosynthesis, characterization, *in vitro* antioxidant, and skin anticancer activity evaluations of the LI aerial parts’ aqueous and hydroalcoholic extract-based zinc and iron NPs. By systematically evaluating the role of extraction protocols and providing quantitative evidence of the enhanced anticancer and antioxidant properties of LI-based green NPs, this work addresses several critical gaps in sustainable anticancer therapies through exploring the potential of green-synthesized Zn and Fe NPs using aqueous and hydroalcoholic extracts of LI aerial parts. These gaps include the need for more sustainable, effective, and safe anticancer agents, as well as the lack of studies tackling how extraction protocols influence the biological and physicochemical properties of green NPs. The findings not only support the potential of green NPs in biomedical applications but also emphasize the importance of tailoring extraction protocols to optimize the properties of the NPs for targeted applications and specific therapeutic outcomes.

## 2 Material and Methods

### 2.1 Chemicals

Methanol (gradient grade for liquid chromatography LiChrosolv Reag., 99.9%) and ethanol (puriss., absolute, 99.8%) were bought from Honeywell, Germany. Iron(III) chloride hexahydrate (ACS reagent, 97%), potassium bromide (anhydrous, free-flow) extra pure, zinc acetate-2-hydrate, folin and ciocalteu’s phenol reagent, aluminum chloride 98%, gallic acid-1-hydrate extra pure, potassium acetate reagent plus ≥99.0%, quercetin, 2,2-diphenyl-1-picrylhydrazyl (DPPH), sodium carbonate, potassium persulfate (99%, ACS reagent), 2,2′-azino-bis(3-ethylbenzothiazoline-6-sulfonic acid (ABTS), and Trolox were obtained from Sigma-Aldrich, Germany.

### 2.2 Preparation of Extracts

Aerial part samples of *L. inermis* (stem, leaf, fruit) were purchased from Al Khaja stores for natural herbs in Al-Ain, United Arab Emirates (UAE) in June 2022. They were dried and crushed using a home blender. The plant materials were utilized for the preparation of hydroalcoholic and aqueous extracts via maceration and decoction, respectively. The decoction process involved heating 278.4 g of plant material in deionized water at 60 °C for 3 h, maintaining continuous stirring at 1500 rpm.^22^ In contrast, the hydro alcoholic extract was produced by macerating 278.4 g of the powdered plant material in 80% ethanol. Final extracts were obtained by filtration followed by evaporation using a Buchi Rotavapor, Germany. Exhaustive extraction for both types of extracts was performed by repeating the described steps multiple times, resulting in the production of total yields of 118.2069 g (42.459%) and 79.9247 g (28.709%) of aqueous and alcoholic crude extracts, respectively.

### 2.3 Synthesis of the Nanoparticles

Green Fe NPs were synthesized utilizing aqueous and 80% ethanolic extracts, employing a previously established method with slight modifications.^23^ Briefly, 20 mg/mL of the extract (100 mL) were combined with 0.02 M FeCl_3_·6H_2_O (300 mL) by mixing 1.6212 g with 300 mL of D.I. H_2_O. The mixture immediately changed color from yellow to black, indicating the formation of Fe NPs. The solution was then refluxed at 55–60 °C with stirring at 500 rpm for 90 min using a Witeg MSH-20D hot plate (Germany) to facilitate the nucleation and growth of the NPs.

**Figure 1:**
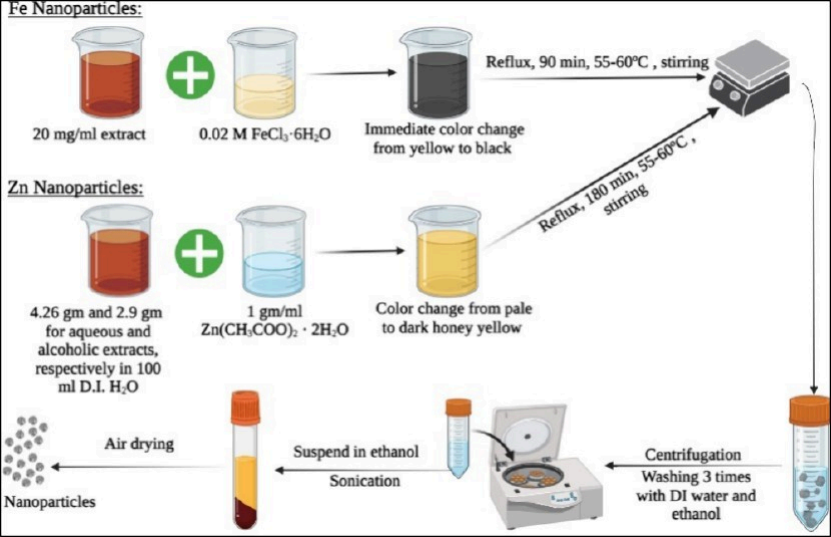
Schematic diagram of the synthesis approach of LI-based metal NPs.

Biosynthesis of ZnO nanoparticles was executed according to Ashraf et al. with minor modifications using both the alcoholic and aqueous extracts.^24^ In the first stage, 1gm/mL zinc acetate dihydrate (10 mL) was mixed with 100 mL of plant extract (2.9 and 4.26 g for alcoholic and aqueous extracts, respectively) and stirred at 500 rpm for 3 h at 55–60 °C. After both reactions were completed, the flasks were allowed to cool. The pellets of the NPs were then collected by centrifugation, and the supernatant was discarded. In the last stage, the pellets containing NPs were washed and centrifuged three times with distilled water and then ethanol at 4000 rpm for 3–5 min, using an Ohaus FC5714 Frontier 5000 Series Multi Pro Centrifuge. The obtained NPs were air-dried, weighed, ground, and stored for further use (Figure 1). Synthesis was carried out at low temperature, and air drying of the NPs was done to avoid decomposition of the extracts’ bioactive constituents that coat the metal NPs.^25^ This coating provides high stability and boosts the biological activity for the NPs. It is worth noting that using two different sorts of extracts allows for evaluating how extraction methods affect the physicochemical and biological properties of the synthesized green NPs, as different solvents extract unique active constituents.^22^ This approach aims to attain the highest quality and potency of NPs.

### 2.4 Characterization of the Nanoparticles

UV–Vis Spectrophotometry, Dynamic Light Scattering (DLS), Scanning Electron Microscope equipped with Energy-Dispersive X-ray Spectroscopy (SEM/EDX), Fourier-transform infrared Spectrophotometer (FT-IR), and X-ray diffraction (XRD) analyses were applied to characterize the biofabricated NPs. DLS gives an idea about the size range of the NPs besides their surface charge and agglomeration state. Likewise, SEM is important for shape, size, agglomeration state, and surface morphology determination, in addition to visualizing capping of the NPs with the organic compounds of the extracts. EDX is conducted to analyze the elemental composition of the produced NPs; in contrast, FT-IR is important for the molecular investigation of the organic compounds that are coating the NPs. Crystallinity and optical properties of the resulted NPs are evaluated using XRD and UV–Vis Spectrophotometry, respectively.^26,27^

#### 2.4.1 Powder X-ray Diffraction (PXRD)

Shimadzu-6100 X-ray diffractometer (XRD), Kyoto, Japan, with Cu Kα radiation of 1.542 Å wavelength was conducted to generate the XRD patterns of the biosynthesized NPs. The data were obtained in a continuous scan at 2θ/θ angle ranging between 10° and 80°, with voltage at 40 kV, current at 30 mA, scanning rate of 1°/min, and step degree of 0.02, with slit conditions of 1.0 divergence, 1.0 scattering, and 0.3 receiving.

#### 2.4.2 Scanning Electron Microscope (SEM) and Energy-Dispersive X-ray Spectroscopy (EDX)

Quattro SEM equipped with an EDX detector of Thermo Fisher Scientific, USA was used to capture images of the final NPs and map their elemental compositions. The instrument was operated at a high vacuum with an accelerating voltage of 10–30 kV and a magnification of 5000–20000×.

#### 2.4.3 Dynamic Light Scattering (DLS)

Dynamic Light Scattering (DLS) Malvern Zetasizer Nano (Malvern Instruments, UK) was employed to determine the mean particle sizes (z-average) and zeta potential. The samples were prepared prior to measurement by dilution with ethanol to obtain an appropriate scattering intensity. The analyses were executed at a flow rate of 0.500 and 25 °C in a measurement position of 4.65 and 2 mm for size and zeta analyses, respectively. The zeta potentials and z-averages of the tested samples were obtained by calculating the average of 3 runs with up to 18 and 126 measurements using clear disposable cells and cuvettes, respectively.

#### 2.4.4 UV-Visible Spectrophotometer

A Cary 60 UV–vis Spectrophotometer (Agilent Technologies Ltd., Malaysia) was utilized to detect the plasmon resonance of the biofabricated NPs through measuring the dispersed NPs in ethanol. The spectral data were recorded in the range of 200–800 nm to detect the characteristic peaks that confirm the formation of Zn and Fe NPs.

#### 2.4.5 Fourier-Transform Infrared (FT-IR) Spectroscopy

A Nicolet NEXUS 470 Fourier-transform infrared (FT-IR) Spectrophotometer (Thermo Scientific, USA) was used to identify the functional groups of the organic phytoconstituents that were involved in the reduction, stabilization, and capping of the NPs, in addition to the determination of the peaks related to the metal itself. The samples were ground and mixed well (0.002 g) with KBr (0.2 g). The mixture was compressed into a thin disc using a hydrolytic compressor. The formed sample discs were exposed to analysis in the range of 400–4000 cm^–1^ with average scans of 64 at 25 cm^–1^ spectral resolution and 15798.3 cm^–1^ laser frequency. The spectrum was recorded in transmittance (%) mode.

### 2.5 Chemical Investigation of Total Phenolic and Flavonoid Contents

#### 2.5.1 Total Phenolic Content (TPC)

Total phenolic contents of the crude extracts and their related phyto-fabricated NPs were estimated following the Folin-Ciocalteu protocol.^28^ This method is based on the ability of phenolics to reduce the Folin-Ciocalteu reagent to form molybdenum–tungsten, which is blue in color and is measured spectrophotometrically. The color intensity increases linearly with the concentration of phenolics in the reaction medium.^29^ In brief, 100 μL of the sample (1 mg/mL) was combined with Folin-Ciocalteu reagent (125 μL) and 750 μL of sodium carbonate solution (15% w/v), and then the final volume was adjusted to 5 mL with D.I. H_2_O and mixed properly. Blank was prepared by following similar steps and replacing the sample with the solvent that was used to solubilize the sample (80% methanol). The resulting mixture was incubated in the dark for 90 min at room temperature and then measured at 765 nm using a Cary 60 UV–vis Spectrophotometer (Agilent technologies Ltd., Malaysia). TPC of the sample is expressed as μg of gallic acid equivalents per milligram of dry weight of the sample (μg of GAE/mg of DW). It is calculated using the resulting average absorbance of the samples and the linear equation of the constructed standard curve of gallic acid (*y* = 0.0023*x* – 0.047). The standard curve was prepared by dissolving gallic acid in 80% methanol in a concentration of 1 mg/mL to prepare a stock solution, which was further diluted into 40, 80, 120, 160, 200, 240, and 280 μg/mL. Thereafter, they were treated according to the above-mentioned steps instead of the sample, and blank was prepared by a similar way and replacing the sample with solvent (80% methanol). The calculated average absorbance was plotted against its concentration. All measurements were carried out in triplicate and expressed as the mean ± standard deviation (SD).

#### 2.5.2 Total Flavonoid Content (TFC)

Total flavonoid contents of the crude extracts of LI and their corresponding green-fabricated NPs were investigated by carrying out an aluminum chloride colorimetric assay.^28^ The basic principle of this method is that the reaction between flavonoids and aluminum chloride leads to the formation of colored acid-stable flavonoid-aluminum complexes with the C-4 keto group and either the C-3 or C-5 hydroxyl group of flavones and flavanols. These complexes can be measured spectrophotometrically.^30^ Briefly, the samples under study were prepared by mixing them with suitable solvent (methanol 80%) with a concentration of 1 mg/mL. Subsequently, the sample (0.5 mL) was mixed with 10% AlCl_3_ (0.1 mL), 1 M potassium acetate (0.1 mL), and 95% methanol (1.5 mL), and then the volume was completed to 5 mL with D.I. H_2_O and mixed thoroughly. The blank was prepared following the same steps, but the sample was replaced with a solvent (80% methanol) only. Then, the mixture was incubated at room temperature for 60 min in the dark. Finally, the absorbance was measured at 415 nm via a Cary 60 UV–vis Spectrophotometer (Agilent technologies Ltd., Malaysia). TFC was expressed as μg of quercetin equivalents per milligram dry weight (μg Qu/mg of DW) of the sample and calculated using the linear equation *y* = 0.0066*x* + 0.0143, of the quercetin standard curve. Preparation of a standard curve of quercetin was done by preparing different concentrations of quercetin (5–100 μg/mL) from a stock solution of quercetin dissolved in 80% methanol in a concentration of 1 mg/mL. Consequently, the prepared concentrations were treated according to the above steps instead of the sample, and the calculated average absorbance is plotted against its concentration. All measurements were performed in triplicate and expressed as the mean ± standard deviation (SD).

### 2.6 Q-TOF LC/MS/MS Analysis of the Extracts

Metabolic profiling of the alcoholic and aqueous extracts of LI was carried out via Quadrupole time-of-flight liquid chromatography with tandem mass spectrometry (Q-TOF LC/MS/MS) to determine their comprehensive phytochemical content.

#### 2.6.1 Sample Preparation

Reconstitution solvent was concocted by using a mixture of distilled water, methanol, and acetonitrile in a 50:25:25 ratio (v/v). Next, 1 mL of the reconstitution solvent was combined with a 50 mg extract; after that, the mixture was vortexed for 2 min, followed by ultrasonication for 10 min, and then centrifugation at 10000 rpm for 10 min. A 50 μL stock was subsequently diluted to 1000 μL with the reconstitution solvent. The final injected concentration was 2.5 μg/μL, and the volume for sample injection was 10 μL. Additionally, a blank sample injection of 10 μL of reconstitution solvent was carried out.^31,32^

#### 2.6.2 Q-TOF LC/MS/MS Analysis and Acquisition Method

The separation of constituents was conducted using an ExionLC system provided by AB Sciex, based in CA, USA. This system was outfitted with In-Line filter disks precolumn (0.5 μm × 3.0 mm, Phenomenex), an X select HSS T3 column (2.5 μm, 2.1 × 150 mm, Waters, USA), and connected to a high-resolution mass spectrometer (Triple-TOF 5600+, AB Sciex, CA, USA). LC-Triple TOF control was managed using Analyst TF 1.7.1 software from Sciex, CA, USA. The analysis employed gradient elution of the mobile phase with a flow rate of 0.3 mL/min to ensure thorough elution of various analytes. The mobile phase consisted of A (+ve mode): 5 mM ammonium formate buffer at pH 3 containing 1% methanol, B (−ve mode): 5 mM ammonium formate buffer at pH 8 containing 1% methanol, and C (+ve/–ve mode): 100% acetonitrile. Elution proceeded as follows: 0–21 min, with 95% of A or B and 5% of C; 21–28 min, with 5% A or B and 95% C; 28.1–35 min, with 95% A or B and 5% C. The injection volume was 10 μL, and the column temperature was set to 40 °C. TOF masses ranging from 50.0000 to 1000.0000 Da were acquired for both MS1 and MS2. High-resolution TOF scanning and Information-Dependent Acquisition (IDA) were executed in MS1 and MS2, respectively. Criteria for switching exceeded 200 cps, former ions were excluded after 3 repeats, former target ions were excluded for 3 s, isotopes were excluded within 2.0 Da, the maximum number of candidate ions to monitor per cycle was 15, and the ion tolerance was set at 10.000 ppm. Dynamic Background Subtraction was applied as well.

#### 2.6.3 Data Analysis and Processing

Master View software was utilized to extract features (peaks) from the Total Ion Chromatogram (TIC) based on specific criteria. These criteria included ensuring that features had a Signal-to-Noise ratio greater than 10, which is typical for nontargeted analysis, and features’ intensities in the sample-to-blank comparison should be greater than 3. For data processing, MS-DIAL 4.9 from the RIKEN Center for Sustainable Resource Science, Japan, and PeakView from Sciex, CA, USA, were employed. Identification was conducted by running the generated data against the RIKEN tandem mass spectral “ReSpect” Databases, both negative (1573 records) and positive (2737 records), as well as by comparing them against published data from the literature and Mass Bank database. The identification score cutoff was set at 70%, and mass error limitation was ensured to be lower than 10 ppm.

### 2.7 Q-TOF LC/MS/MS Based Multivariate Data Analyses and Fingerprinting of Henna Extracts

Principal Component Analysis (PCA) and Orthogonal Projections to Latent Structures Discriminant Analysis (OPLS-DA) was constructed according to El-Hawary et al. for the LC-MS-MS data of the alcoholic and aqueous extracts of LI using the Metware Cloud, a free online platform for data analysis (https://cloud.metwarebio.com).^33^ This was done to determine the separations between them and the marker compounds in each of them, which would affect the physicochemical and biological behaviors of their corresponding green synthesized NPs.

### 2.8 Evaluation of Antioxidant Activity (*In Vitro*)

The antioxidant effect of the NPs was investigated via DPPH and ABTS colorimetric radical scavenging assays following specific protocols with some modifications.^33−37^ In the DPPH assay, stock solutions of positive control (TROLOX) and the samples were prepared by mixing 200 μg of each with 2 mL of 80% methanol. Subsequently, from the prepared stock solutions, 200 μL was introduced into 96-well plates in 6 different concentrations (10, 20, 40, 60, 80, 100 μg/mL) and mixed with 100 μL of DPPH, then left in darkness for 40 min at 24 °C. Methanol 80% mixed with DPPH was utilized as the negative control, while methanol 80% only was used as the blank. In contrast, a sample blank consisting of the sample dispersed in 80% methanol was applied for each concentration of the tested NPs due to their dispersion in the solvent and their poor solubility. Finally, the plates were measured to get the absorbance at 517 nm via a UV microplate reader (Hidex Sense, Hidex, Turku, Finland).

The percentage radical scavenging effect of the samples understudy was calculated using the following formula:
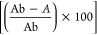
where Ab represents absorbance of the −ve control, while *A* is for the sample’s absorbance. Calculation of the IC50 (μg/mL) values was done by plotting a calibration curve in the linear range for each tested sample. This was achieved by plotting the sample concentration against its corresponding %radical scavenging. IC50 is the concentration required by the samples to decrease DPPH absorption by 50%; thus, the lower the IC50, the higher the antioxidant activity of the sample. In the ABTS radical scavenging assay, stock solutions of the samples and positive control (TROLOX) under investigation were prepared with concentration of 100 μg/mL in 80% methanol. From the stock solutions, the following concentrations were prepared; 10, 20, 40, 60, 80, and 100 μg/mL; to be tested. To form the ABTS+· radical cation, 7 mM of ABTS was mixed with 2.45 mM potassium persulfate in a ratio of 1:1, then the mixture was incubated for 12–16 h in a dark room. The mixture was then diluted with methyl alcohol until the absorbance value reached 0.70 ± 0.02 at 734 nm. Consequently, in a 96-well plate, each sample concentration solution (100 μL) was mixed with 100 μL of the diluted ABTS+·, then incubated in darkness for 10 min at room temperature. Eventually, the absorbance of the plate was recorded at 734 nm via Hidex Sense UV microplate reader (Hidex,Turku, Finland). Methanol 80% with ABTS+· was utilized as the negative control, whereas methanol 80% only was applied as the blank. In contrast, due to the poor solubility and uniform dispersion of the tested NPs in the solvent, a sample blank was employed for each concentration. This blank consisted of NPs dispersed in 80% methanol.

The % radical scavenging effect is calculated according to the following formula:
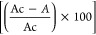
where Ac is the absorbance of the negative control, and *A* is the absorbance of the sample. The IC_50_ values (μg/mL) were then calculated by using a calibration curve in the linear range by plotting the extract concentrations against their related percentages of radical scavenging. Likewise, for DPPH assays, the higher the IC_50_ value, the lower the antioxidant activity of the sample, as it represents the required concentration by the sample to decrease ABTS absorption by 50%. All the measurements were done in triplicates and expressed as mean ± standard error (SE).

### 2.9 Investigation of Skin Anticancer Activity (*In Vitro*)

#### 2.9.1 Cell Culture

Cell lines derived from A-431 human epidermoid skin carcinoma (skin/epidermis) were obtained from Nawah Scientific Inc., located in Mokatam, Cairo, Egypt. These cell lines were cultured in DMEM medium, which was supplemented with 100 mg/mL streptomycin, 100 units/mL penicillin, and 10% heat-inactivated fetal bovine serum. The maintenance of the cell lines was carried out in a humidified 5% (v/v) CO_2_ atmosphere at 37 °C.

#### 2.9.2 Cytotoxicity Assay

Cell viability was determined through the Sulforhodamine B (SRB) assay. In 96-well plates, 100 μL aliquots of cell suspension (5 × 10^3^ cells) were plated and incubated in complete media for 24 h. Subsequently, cells were exposed to drugs at various concentrations by treating them with another 100 μL aliquot of media containing drug. After 72 h of drug treatment, cell fixation was done through the replacement of the media with 150 μL of 10% trichloroacetic acid (TCA), which after that incubated for 1 h at 4 °C. After removing TCA, cells were washed five times with distilled water. Afterward, 0.4% w/v SRB solution (70 μL) was introduced to the plates and incubated at room temperature in darkness for 10 min. Plates underwent washing 3 times with 1% acetic acid and were air-dried overnight. Next, 10 mM of TRIS (150 μL) was used to dissolve the protein-bound SRB stain, followed by measuring the absorbance at 540 nm via a BMG LABTECH-FLUOstar Omega microplate reader (Ortenberg, Germany).^38,39^

## 3 Results and Discussion

### 3.1 Characterization of the Nanoparticles

The resulting LI-based NPs exhibit distinct physical properties. Consequently, we utilized various techniques to analyze their physicochemical characteristics. HZnC and HFeC refer to the zinc and iron NPs synthesized with the alcoholic extract, respectively, while HZnQ and HFeQ denote the zinc and iron NPs synthesized by using the aqueous extract, respectively.

#### 3.1.1 Powder X-ray Diffraction (PXRD)

The crystalline nature, structure, and phase composition analysis of the biosynthesized LI-mediated NPs, using the XRD, indicates that they are amorphous with poor crystalline structure (Supporting Information: S1).

#### 3.1.2 Scanning Electron Microscope (SEM) and Energy-Dispersive X-ray Spectroscopy (EDX)

**Figure 2:**
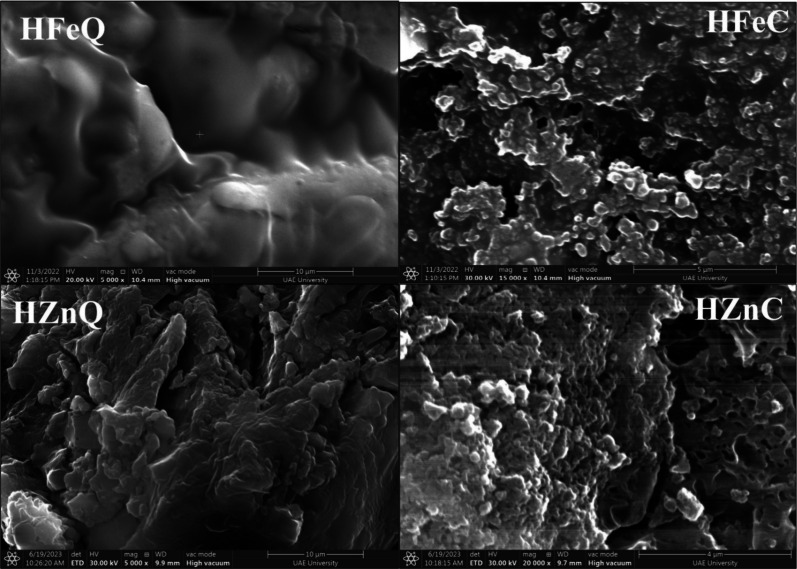
SEM images of Zn and Fe NPs using both aqueous and 80% ethanolic extracts.

Surface morphology and agglomeration state of the biofabricated NPs were visualized via SEM. Figure 2 shows a fluffy irregular-like shape of the surface, displaying a higher tendency of agglomeration in the case of HFeQ and HZnQ than those prepared using the alcoholic extracts. This reveals that alcoholic extract may be better than the aqueous in capping of the NPs. It was frequently reported that the green synthesized NPs showed a high tendency of agglomeration due to the adhesive nature of the utilized plant extracts.^4,23,40−44^ HFeC NPs demonstrated the best uniformity, dispersion, and homogeneity among the other formed NPs.

**Figure 3:**
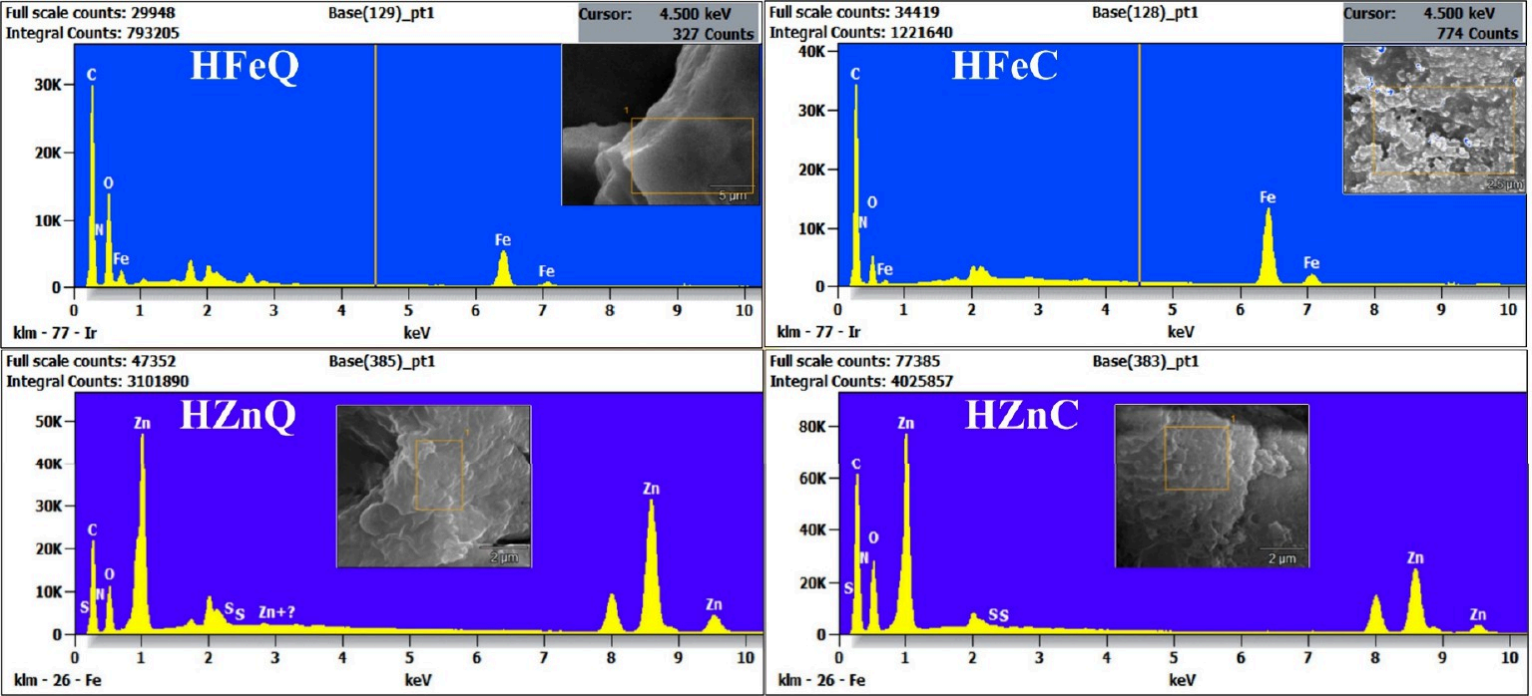
EDX analysis of the phyto-synthesized NPs.

**Figure 4:**
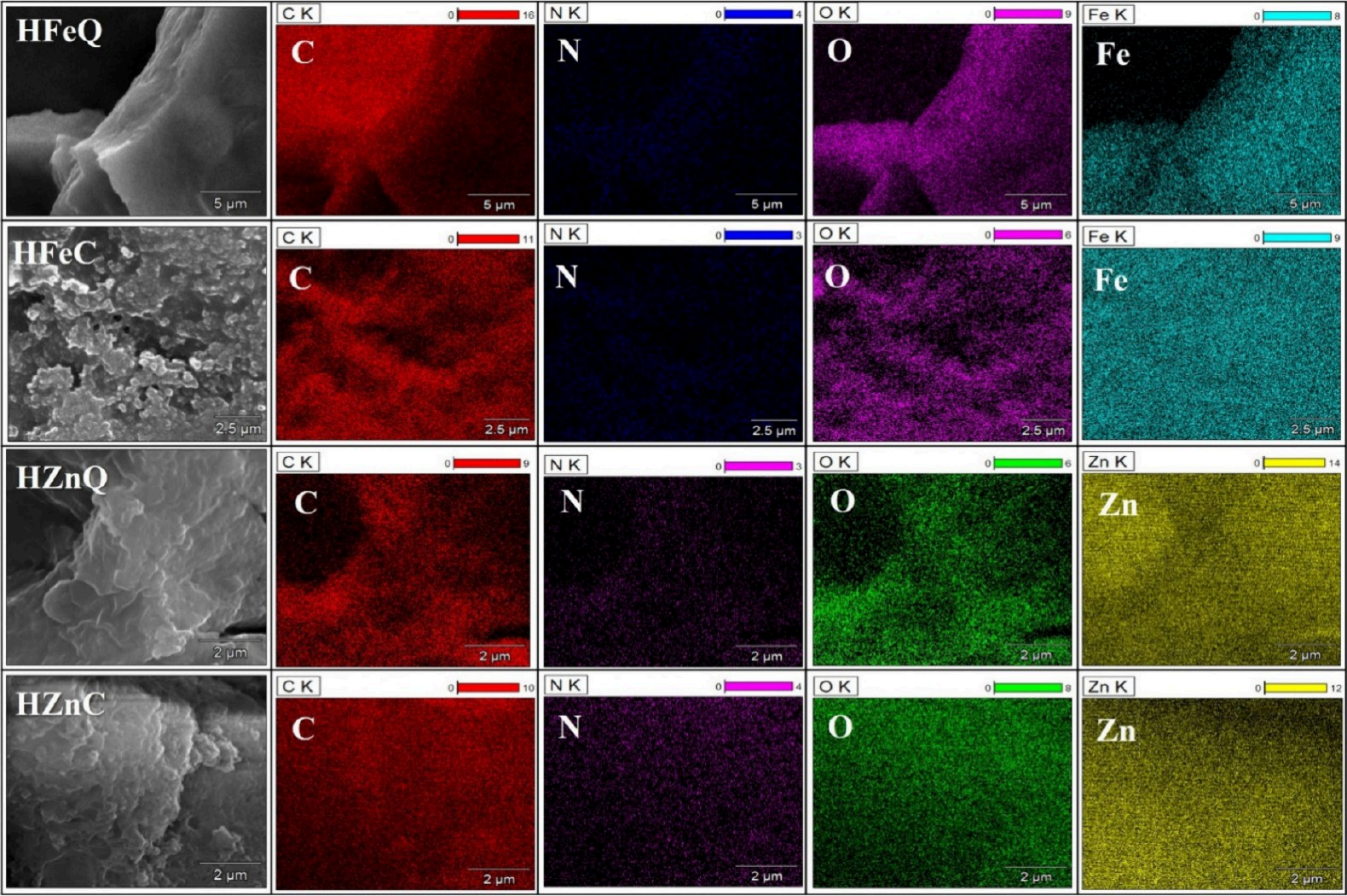
Elemental mapping of the LI-based NPs.

EDX analysis was carried out to confirm the formation of Fe and Zn NPs and investigate the elemental composition of their coating. The recorded peaks of iron and zinc confirm the formation of the Fe and Zn NPs. The results revealed that the average weights of iron in the analyzed samples were 27.67% and 31.88% for HFeC and HFeQ NPs, respectively, and 26.1% and 53.03% for HZnC and HZnQ NPs, respectively. Other peaks for carbon and oxygen were shown between 0.1 and 0.5 keV with 36.29%, 43.66%, 47.9%, and 33.68% for oxygen and 43.98%, 34.37%, 38.63%, and 28.53% for carbon in HFeC, HFeQ, HZnC, and HZnQ NPs, respectively. Trace amounts of nitrogen, ranging between 1.87% - 5.21%, were detected in HFeC, HZnQ, and HZnC. These observed other elements like sulfur are linked to the plant phytoconstituents that are coating the NPs (Figures 3 and 4). Analysis reveals that NPs synthesized by using aqueous extracts exhibit higher concentrations of iron (Fe) and zinc (Zn) compared to those produced with alcoholic extracts. This observation suggests that aqueous extracts possess stronger reducing capabilities than alcoholic extracts. In contrast, the amount of C element is higher in the alcoholic-derived NPs than in the aqueous-mediated ones. Thus, we hypothesize that the phytochemicals present in the alcoholic extract may possess properties that enable them to form a more effective coating around the nanoparticles compared with those in the aqueous extract. This observation aligns with the SEM images, indicating a reduced propensity for agglomeration in the NPs based on the alcoholic extract. It can be inferred that this is attributed to their more abundant coating and capping with phytochemicals, providing greater stability and dispersion for the alcoholic-extraction-based NPs compared to those based on aqueous extract.

#### 3.1.3 Dynamic Light Scattering (DLS)

**Figure 5:**
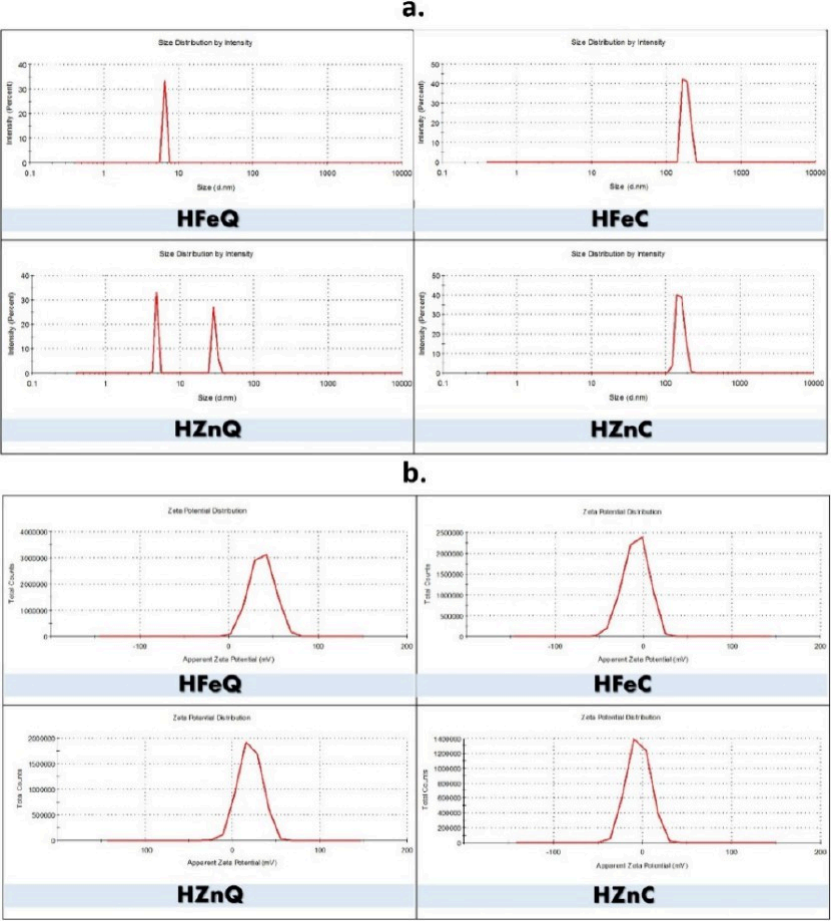
(a) Size distribution and (b) zeta potential distribution of the formed LI-based NPs.

Employing a Malvern Zetasizer DLS, we determined the hydrodynamic diameter, zeta potential, and polydispersity indices (PDIs) of the bioformed NPs dispersed in ethanol at ambient temperature. Zeta potential serves as an indicator for the stability of nanoparticles because it is correlated with the stability of colloidal dispersions by reflecting the extent of repulsion between the neighboring particles that carry similar charges in the dispersion. The higher the zeta potential value, the stronger the stability of the particles. Moreover, polydispersity index is important for the determination of homogeneity of the particles’ sizes since the higher its value the greater the heterogeneity among the particles.^45^ The resulting data are presented in Table 1. Also, distribution graphs of particle sizes and zeta potentials for all of the tested NPs are shown in Figure 5, respectively. According to the given values, HFeQ exhibited the smallest particle size (6.503 nm), showing the highest monodispersity and stability with PDI and zeta potential values of 1.68 × 10^–16^ and 36.7 mV, respectively. HZnQ is the second-best one in terms of the three parameters followed by HFeC and HZnC. This indicates that the aqueous extract-based NPs are better than the alcoholic-based ones in regards to particle size, monodispersity, and stability.

**Table 1:** Mean Particle Size, Zeta Potential Values, and Polydispersity Indices of the Synthesized Green NPs According to NNLS Fitting

NP	Size (d.nm)	Zeta potential (mV)	PDI
HFeC	184.1	–8.68	0.012
HFeQ	6.503	36.7	1.68 × 10^–16^
HZnC	158.3	–4.32	0.015
HZnQ	4.844, 29.04	20.1	0.0001, 0.004

#### 3.1.4 UV Visible Spectrophotometer

Surface plasmon resonance of LI-coated Fe and Zn NPs was confirmed by their UV–vis absorption spectra (Supporting Information S2). Characteristic absorption peaks at 265.1 and 269.9 nm in HFeC and HFeQ NPs samples, respectively, denote the formation of Fe NPs. Moreover, the presence of Zn in HZnC and HZnQ samples was confirmed by the optical transitions that were observed at 270 nm. Iron and zinc NPs typically display a broad absorption band in the UV–vis region. For iron NPs, this band is generally observed between 220 and 288 nm, while for zinc NPs, it falls within the range of 230 to 330 nm.^23,46,47^

#### 3.1.5 Fourier-Transform Infrared (FT-IR) Spectroscopy

**Figure 6:**
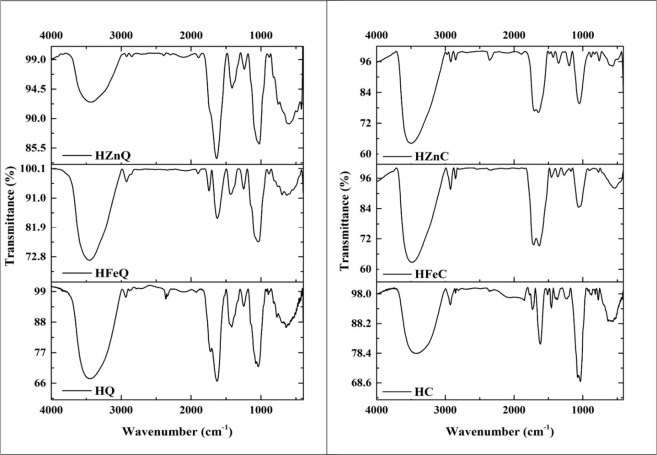
FT-IR spectra of the fabricated green NPs.

FT-IR analysis is a reliable strategy that was conducted to investigate the molecular composition of the extracts under study and the surface functionalization of their corresponding Fe and Zn NPs. The generated spectra of the measured samples are listed in Figure 6. The broad bands at the range of 3426–3496 cm^–1^ indicate O–H stretching corresponding to carboxyl acid and alcoholic hydroxyl groups, while the low and narrow peaks at the range of 2849–2933 cm^–1^ signify C–H stretching. The bands between 2105–2390 cm^–1^ are for C≡N and C≡C vibrations. The existence of C=O and C=C functional groups is confirmed by the appearance of strong bands in the vicinity of 1616–1892 cm^–1^. The vibrational bands that present between 1352–1513 cm^–1^ represent nitro compounds. The spectra at 1024–1269 cm^–1^ authenticate the presence of C–O vibrations. Several bands can be observed at lower frequencies around the range of 530.29–903.15. These bands are related to halogenic compounds, aromatic rings, and organometallics (Zn–O and Fe–O).^48^ Accordingly, the FTIR spectra of the analyzed samples designate the presence of alcoholic, phenolic, carbonyls, alkanes, alkenes, alkynes, alkyl, nitro, halides, and aromatic functional groups. This could be attributed to the presence of various phytochemical classes, such as phenolics, flavonoids, fatty acids, amino acids, saponins, quinones, and terpenoids, in the samples, and this confirms the excellent capping of the NPs with the plant chemical content. Conferring to the findings of this analysis, the FT-IR spectra of the LI extracts and their corresponding NPs are close to each other, which verify the positive green fabrication of the plant-based iron and zinc nanoparticles proposed in this study. The absence of CΞN and CΞC peaks in the IR spectrum of HFeQ suggests that the phytochemicals that possess these functional groups could be completely consumed in the reduction process of the metal precursor. Our results corroborate findings from earlier research.^23,49^

### 3.2 Chemical Investigation of Total Phenolic and Flavonoid Contents

#### 3.2.1 Total Phenolic and Flavonoid Contents

Phenolic and flavonoid compounds are considered as the main classes of secondary metabolites in almost all of the plants’ species in general. They possess several significant roles in the synthesis of NPs through the reduction of metal precursors. They were proven to be strong reducing and efficient capping and stabilizing agents that exhibit an exceptional ability to control the relative rates between nucleation and growth to reach the desired metal NPs for the aimed applications.^50−52^ Moreover, they are well-known for their potency against a broad range of diseases, such as being effective antioxidant and anticancer agents due to their redox properties.^53^ Thus, plants’ strong reducing capability, antioxidant, and anticancer effects are strongly assumed to be directly related to their phenolic and flavonoid contents. Therefore, we carried out phenolic and flavonoid content investigations of the plant under study and its corresponding green NPs. It is clear from the results (Table 2) that the crude alcoholic extract contains the highest content of phenolics (90.22 ± 1.51 μg of GAE/mg of DW), followed by the aqueous extract (82.51 ± 1.73 μg of GAE/mg of DW), then the alcoholic-based Fe and Zn NPs. In contrast, the aqueous-based NPs showed the lowest content of phenolics among the tested samples. Since the phenolic content is higher in the crude extracts than their mediated NPs, this means that part of this phenolic content contributed mainly to the reduction of the metal precursor during the NPs’ synthesis process. Unlike TPC, the crude extracts have shown lower TFC than their related NPs. HZnC exhibited the highest flavonoid content (30.48 ± 0.83 μg of Qu/mg of DW), followed by HFeC (24.75 ± 0.50 μg of Qu/mg of DW) (Table 2). Saad et al. provided an explanation for this phenomenon, stating that certain polyphenolic compounds, when involved as reducing agents in the NPs’ synthesis, oxidation of the phenol group takes place to form quinoid compounds. These compounds are then adsorbed onto the surface of the NPs, leading to an increase in their phenolic content and subsequent stabilization.^54^ On the other side, the aqueous extract-mediated NPs demonstrated the lowest flavonoid contents, and they were lower than those of their corresponding extract, which means that the flavonoid contents in the aqueous extract directly participated in the reduction of both metal ions.

**Table 2:** Total Phenolic and Flavonoid Contents of the Bio-formulated Green NPs and Their Corresponding Extracts^a^

	HC	HQ	HZnC	HFeC	HZnQ	HFeQ
TPC (μg of GAE/1 mg of DW)	90.22 ± 1.51	82.51 ± 1.73	39.57 ± 2.52	55.13 ± 2.28	36.22 ± 4.16	29.33 ± 2.94
TFC (μg of QU/1 mg of DW)	20.50 ± 0.57	16.66 ± 2.99	30.48 ± 0.83	24.75 ± 0.50	7.10 ± 1.18	2.49 ± 0.74

a Results are presented as means ± SD (n = 3).

### 3.3 Q-TOF LC/MS/MS Analysis of the Extracts under Study

A Q-TOF LC/MS/MS investigation was applied to obtain a comprehensive report on the phytochemical contents of both alcoholic and aqueous extracts of LI. A thorough elution of metabolites in negative and positive ionization modes was achieved as demonstrated in the resulted total intensity and base peak chromatograms (Supporting Information S3–S10). In total, 94 metabolites were tentatively identified in the negative mode, and 87 compounds were determined in the positive mode. These phytochemicals belong to several chemical classes, including coumarins, flavonoids, phenolic acids, amino acids, hydrocarbons, terpenes, and fatty acids. The retention times and fragmentation patterns of the characterized compounds, along with their identifications, are provided in Tables 3 and 4. Based on the acquired data, phenolic compounds represent the largest share of the identified compounds since 44 phenolic phytochemicals were detected in the negative mode out of a total of 94 compounds, while 37 phenolics were identified out of 87 compounds in the positive mode. Sixty of these phenolics were detected in HC, while 55 were identified in HQ. The following phenolics were detected in both extracts; myricetin, 3-(4-Hydroxy-3-methoxyphenyl)prop-2-enoicacid, syringetin-3-O-glucoside, syringetin-3-O-galactoside, luteolin-6-C-glucoside, apigenin 8-C-glucoside, E-4,5′-dihydroxy-3-methoxy-3′-glucopyranosylstilbene, kaempferol-7-neohesperidoside, E-3,4,5′-trihydroxy-3′-glucopyranosylstilbene, quercetin-4′-glucoside, maritimetin-6-O-glucoside, daidzein-8-C-glucoside, apigenin-7-O-glucoside, eriodictyol-7-O-glucoside, phlorizin, 3′-methoxy-4′,5,7-trihydroxyflavonol, rhoifolin, 4-deoxyphloridzin, kaempferol-3-O-alpha-L-rhamnoside, 3′,4′,5,7-tetrahydroxyflavanone, apigenin, acacetin, 3,5,7-trihydroxy-4′-methoxyflavone, esculin, 3,3′,4′,5-tetrahydroxy-7-methoxyflavone, procyanidin B2, 4′-hydroxyisoflavone-7-O-glucoside, kaempferol-3-O-rutinoside, hyperoside, sinapyl aldehyde, genistein, ononin, cyanidin-3-glucoside, and coumaric acid. In contrast, 21 phenolic compounds were tentatively annotated in HQ only. Also, isorhamnetin-3-O-glucoside, xanthine, isoquercitrin, taxifolin, pelargonidin-3-O-glucoside, eriodictyol-7-O-neohesperidoside, daidzein, okanin-4′-O-glucoside, hesperetin, daphnetin, naringenin, kynurenic acid, 7-acetoxy-4-methylcoumarin, quercetin, sissotrin, chalcone, cyanidin-3,5-di-O-glucoside, peonidine-3-O-glucoside chloride, riboflavin, 3,4-dimethoxycinnamic acid, 3-(4-hydroxy-3,5-dimethoxyphenyl)-2-propenoic acid, cyanidin-3-O-rutinoside, luteolin, kaempferol-3-O-(6-p-coumaroyl)-glucoside, malvidin-3-galactoside, and 1-O-*b*-d-glucopyranosyl sinapate were exclusively determined in HC. It is lucid from the results that the number of the identified phenolics is greater in the negative ionization mode than the positive one. Due to their weakly acidic nature and high p*K*_a_ values, phenolic compounds preferentially undergo ionization in negative electrospray ionization (ESI) mode.^55,56^ The generated data showed a common loss of pentosyl (132 amu), rhamnosyl (146 amu), and hexosyl (162 amu) sugar units in flavonoid glycosides. Other common losses were recorded, such as the loss of water molecule (18 amu) and CH_3_ (15 amu), due to the presence of methyl and hydroxyl groups in phenolic compounds. For example, in the negative ionization mode [M-H]^−^, peak 35 of syringetin-3-O-glucoside was observed at *m*/*z* 507.1339 (C_23_H_24_O_13_)^−^, which gave specific fragments at *m*/*z* 345.08229 due to the loss of hexosyl sugar [M-H-hexoside]^−^. Likewise in peak 58 at *m*/*z* 431.0973 [M-H]^−^, which gave distinct fragments at *m*/*z* 269.03954 due to the loss of hexosyl [M-H-162]^−^, beside *m*/*z* 161.02502:592 due to the loss of the aglycone [M-H-270]^−^; thus, it was identified as apigenin 8-C-glucoside. Also, in peak 59 at *m*/*z* 463.0865, a characterized fragment at *m*/*z* 301.03656 was detected due to the loss of hexosyl sugar unit [M-H-162]^−^, beside another fragment at *m*/*z* 271.02818 due to the cleavage of ring B and the loss of hexosyl sugar unit [M-H-162-C_1_H_2_O_1_]^−^; therefore, it was identified as Isoquercitrin. The same was observed in the positive ionization mode in peak 16 at *m*/*z* 451.1337, at which distinct fragments were observed at *m*/*z* 289.11219 [M-H-162]^−^ and *m*/*z* 273.07558 [M-H-162-O_1_]^−^ due to the loss of hexosyl unit and oxygen. In peak 55 at *m*/*z* 595.1657, a loss of hexosyl and ramnosyl sugar units were detected [M+H-146–162]^+^ in a fragment at *m*/*z* 287.06074, beside another distinguished fragment at *m*/*z* 147.06989 due to the loss of the aglycone and hexosyl sugar unit [M+H-286–162]^+^, and a fragment at *m*/*z* 577.16782 due to the loss of water [M+H-18]^+^. Therefore, this peak was assigned as kaempferol-3-O-rutinoside. For peak 58, which was ascribed as hyperoside at *m*/*z* 465.1002, two major peaks were observed at *m*/*z* 303.05938 [M+H-162]^+^ and 257.05283 [M+H-162- ring B cleavage C_1_H_2_O_2_]^+^. Flavonoid aglycones showed characteristic fragments due to cleavage of rings a, b, or c. An eminent example of this is peak 7 at *m*/*z* 317.0564 as it was ionized in the negative mode to give two distinctive fragments at *m*/*z* 153.02081 due to cleavage of rings a and b, as well as *m*/*z* 273.04526 due to cleavage of ring b. Losses of water and CO molecules were detected in several flavonoids like in peak 47 at *m*/*z* 303.0463, which produced fragments at *m*/*z* 285.13529 [M+H–H_2_O_2_]^+^ and *m*/*z* 256.93034 [M+H–H_2_O_2_–CO]^+^. Phenolic acids demonstrated significant losses of CO_2_ in negative mode (44 amu), COOH in positive mode (45 amu), and H_2_O (18 amu). For instance, peak 26 and 27 at *m*/*z* 163.0403 and 193.0508 [M-H]^−^ showed distinct fragments at *m*/*z* 119.04981 and 148.95689 → representing cinnamic acid moiety, [M-H-44]^−^ due to the loss of CO_2_, respectively. Also, another major fragment at *m*/*z* 134.03754 was observed in peak 27 due to the loss of CO_2_ and CH_3_ [M-H-44–15]^−^. Therefore, those 2 peaks were identified as 3-(4-Hydroxyphenyl)prop-2-enoic acid and 3-(4-Hydroxy-3-methoxyphenyl)prop-2-enoicacid, respectively. Also, in peak 39, which was identified as caffeic acid, at *m*/*z* 179.035 [M-H]^−^, daughter fragments at 135.04481 [M-H-44]^−^ and 116.95741 [M-H-44–18]^−^ were produced. Characteristic fragments at *m*/*z* 191.03696 representing the deprotonated quinic acid moiety due to the loss of the caffeoyl moiety [M–H-C_9_H_6_O_3_]^−^, 147.05461 [M-quinic acid-CH_3_]^−^, 290.89474 [M-H–CO_2_–H_2_O]^−^, and 309 [M-H-44]^−^ were observed in peak 48 at *m*/*z* 353.088, so it was nominated as chlorogenic acid. Moving to peak 36 in the positive ionization mode, which was detected at *m*/*z* 179.0344 (C_10_H_10_O_3_)^+^ and assigned as 4-methoxycinnamic acid, it produced two distinguishing MS^2^ peaks at *m*/*z* 133.03344 [M-COOH]^+^ due to the loss of carboxylic acid moiety, in addition to other peaks at *m*/*z* 105.04069 [M-C_2_HO_3_]^+^. Dimethoxycinnamic acid was also characterized in peak 70 at 209.1157 through its MS_2_ data at *m*/*z* = 135.02405 [M+H–COOH-C_2_H_5_]^+^ and 149.0252 [M+H–COOH–CH_3_]^+^. Coumaric acid was also detected in peak 71 at *m*/*z* 165.1283, exhibiting characteristic MS^2^ fragments at *m*/*z* 147.04584, representing deprotonated cinnamic acid due to the loss of the hydroxyl group [M–OH]^+^ and another major fragment at *m*/*z* 120.95745 [M+H–COOH]^+^ due to the loss of the carboxylic acid group. On the other hand, peak 86 with molecular ion at *m*/*z* 387.1773 (C_17_H_22_O_10_)^+^ was tentatively determined as 1-O-*b*-d-glucopyranosyl sinapate due to the generated MS^2^ fragments at *m*/*z* 93.06819 via the loss of hexose, CO_2_, C_2_H_2_, and 2 methoxy moieties [M+H-162–44–26–62]^+^ and *m*/*z* 119.05465 due to the loss of hexose, CO_2_, and 2 methoxy moieties [M+H-162–44–62]^+^.

**Table 3:** Metabolites Tentatively Identified Using Q-TOF LC/MS/MS in the Alcoholic (HC) and Aqueous Extracts (HQ) of the Aerial Parts of LI in Negative Ionization Mode

										*L. inermis*	
No.	Identification	RT (Min.)	Precursor (*m*/*z*) M^–^	Height	Area	Error (PPM)	Formula	Chemical class	Fragmentation	HC	HQ
1	Malic acid	1.053167	133.0145	138139.6	885251.9	–0.2	C4H6O5	Beta hydroxy acids and derivatives	115.00325:2998, 71.01378:2368, 72.99346:1367, 114.99871:884, 89.02411:300	√	√
2	Maleic acid	1.053167	115.0029	25746.22	163670.1	0.7	C4H4O4	Dicarboxylic acids and derivatives	71.01854:71	√	√
3	Citramalic acid	1.053167	147.0297	5389.111	28107.97	0.9	C5H8O5	Hydroxy fatty acids	103.0425:143, 129.01986:214	√	√
4	Alpha-d-glucose-1-phosphate dipotassium salt dihydate	1.07575	259.0256	3029.222	26478.23	–9.9	C6H13O9P	Monosaccharide phosphates	78.96121:254, 96.96602:143, 154.98709:143, 258.96313:144, 259.02676:440		√
5	Glycerol-2-phosphate	1.079167	171.0035	1930.889	12210.71	5.8	C3H9O6P	Glycerophosphates	78.95742:291, 171.02286:143	√	
6	Inosine	1.1284	267.0722	26769.67	126722.1	0.9	C10H12N4O5	Purine nucleosides	59.01371:405, 71.01215:294, 85.03058:326, 89.02895:214, 113.02574:479, 249.07097:214, 267.07312:1977		√
7	Myricetin	1.131667	317.0564	51857	263747.6	–4.7	C15H10O8	Flavonol	153.02081:107, 273.04526:71	√	√
8	Citrate	1.131667	191.0212	18140.45	174738.6	–1.3	C6H8O7	Tricarboxylic acid	85.0308:325, 87.00994:473, 102.97656:147, 111.01115:799, 146.95719:214	√	√
9	Gluconate	1.131667	195.0496	192853.6	987477.4	5.1	C6H12O7	Medium-chain hydroxy acids and derivatives	57.03547:384, 58.00696:267, 59.01485:1589, 71.01461:879, 72.99189:569, 75.00856:5522, 85.0281:566, 87.01248:774, 89.02374:607, 99.00839:848, 129.01816:3875, 159.02539:746, 177.03277:801	√	√
10	Glyceric acid	1.141567	105.0194	27372.45	150248.7	–0.2	C3H6O4	Sugar acids and derivatives	59.01697:107, 72.99663:250, 105.01851:324		√
11	Thymine	1.1804	125.0247	87100.55	393012.9	–3	C5H6N2O2	Hydroxypyrimidines	51.02434:143, 69.03533:143, 79.01901:293, 81.03534:143, 97.03425:107, 124.01779:331, 125.0264:1869		√
12	L-5-Oxoproline	1.184333	128.0351	33528.67	150571.6	0.3	C5H7NO3	Alpha amino acids and derivatives	82.03503:71	√	
13	Maltitol	1.22315	343.1234	77346.55	412418.4	1.3	C12H24O11	Fatty acyl glycosides of mono- and disaccharides	59.01587:482, 71.01334:338, 89.02498:491, 113.02581:255, 119.03737:437, 161.04227:250, 179.05268:879, 181.07854:368	√	
14	Mannitol	1.2355	181.0706	566353.4	3293358	6.1	C6H14O6	Sugar alcohols	55.01854:1740, 58.00689:1760, 59.0137:11610, 73.03043:2216, 83.01375:1363, 85.028:1502, 89.02498:5611, 101.02515:5165, 113.02431:911, 119.03429:1608, 163.06301:2473	√	√
15	Citraconic acid	1.245233	128.9603	15671.11	89398.41	–3	C5H6O4	Methyl-branched fatty acids	56.9759:36, 57.03668:36, 58.99438:36, 69.0024:36, 85.02817:36, 85.02947:71, 99.00702:36, 100.2786:36, 101.01819:36, 128.99564:36, 129.03734:36		√
16	Galactinol Dihydrate	1.251983	341.1124	32189.22	267015.5	–7	C12H22O11	Disaccharide	135.04827:1742, 161.04625:587, 179.03419:2609	√	
17	L-beta-Homolysine	1.322067	159.0295	6095.889	41688.37	–0.1	C7H16N2O2	Beta amino acids and derivatives	70.99924:642, 71.00162:523, 114.9908:479, 158.97545:180		√
18	D(−)-Gulono-gamma-lactone	1.328317	177.0397	46312.22	261876.8	3.6	C6H10O6	Gamma butyrolactones	57.03249:440, 59.01508:621, 71.01484:873, 85.02963:327, 99.00863:411, 129.01679:1066	√	√
19	Maltotriose	1.34165	503.1477	10995.56	38330.09	–9.3	C18H32O16	Oligosaccharides	163.03636:671, 193.04743:328, 325.09844:287	√	
20	Hinokitiol	1.34165	163.0624	9429.889	54273.96	–6.3	C10H12O2	Terpenoid	119.04676:179	√	
21	3,4-Dihydroxybenzoic acid	1.34165	153.0198	27292.55	92441.03	–0.1	C7H6O4	Hydroxybenzoic acid derivatives	81.95443:114, 82.75802:153, 83.01389:1698, 109.03053:651	√	√
22	Salicylic acid	1.347717	137.0229	6984.222	26465.21	9.9	C7H6O3	Salicylic acids	65.04168:71, 77.01307:72, 81.03392:72, 92.02817:73, 93.03619:331, 137.01958:143		√
23	Methylsuccinic acid	1.399883	130.9667	4864.889	20954.99	0.4	C5H8O4	Methyl-branched fatty acids	86.99292:179		√
24	2,5-Dihydroxybenzoic acid	1.613633	153.0181	5715.556	82690.19	7.3	C7H6O4	Hydroxybenzoic acid derivatives	83.01417:250, 108.02302:250, 109.03377:179	√	√
25	Gibberellin A4	1.6435	331.0659	55410.33	402738.8	1.3	C19H24O5	C19-gibberellin 6-carboxylic acids	59.0141:161, 125.0202:596, 167.99995:179, 168.00727:268, 169.01007:1709, 211.02084:1065,241.03096:179, 271.03932:1179, 331.06817:2561		√
26	3-(4-Hydroxyphenyl)prop-2-enoic acid	2.8175	163.0403	28299.78	263034	–1	C9H8O3	Hydroxycinnamic acids	76.97474:136, 77.04043:402, 94.97928:317, 119.04981:2394, 163.04316:329		√
27	3-(4-Hydroxy-3-methoxyphenyl)prop-2-enoicacid	3.526133	193.0508	3456.667	26999.34	–0.5	C10H10O4	Hydroxycinnamic acids	134.03754:426, 178.02462:183, 149.06205:76, 137.02655:31, 133.03224:58, 117.03238:22, 89.04133:18, 135.0335:14, 148.95689:13, 121.03219:13	√	√
28	L-beta-Homoisoleucine	4.184	144.0446	4735.444	59380.43	1.3	C7H15NO2	Beta amino acids and derivatives	56.99517:36, 83.01277:36, 99.95236:36, 100.95877:71	√	√
29	Kynurenic acid	4.223917	188.0346	9336	75731.52	3.7	C10H7NO3	Quinoline carboxylic acids	143.58701:93, 144.04429:3042, 145.04777:107, 188.03724:322		√
30	(+)-3 3′ 4′ 5 7-Pentahydroxyflavan	4.3439	289.0332	142395.7	596204.6	7.4	C15H14O6	Catechins	186.99849:2376, 187.00429:3610, 201.05577:2442, 202.02816:2336, 203.0351:1284, 245.04436:7837, 289.03351:4860		√
31	P-Hydroxybenzoic acid	4.478483	137.025	4521.333	27182.9	–3.5	C7H6O3	Hydroxybenzoic acid derivatives	65.04066:73, 93.03365:295, 136.92896:71	√	√
32	L-tryptophan	4.841133	203.0352	2483.333	18400.63	–0.7	C11H12N2O2	Indolyl carboxylic acids and derivatives	116.02926:71, 173.02347:54	√	
33	Sebacate	4.894033	201.0557	5232	24972.25	0.8	C10H18O4	Medium-chain fatty acids	138.93754:71, 154.96444:71, 156.94326:71, 158.0279:107, 173.05665:250, 182.94781:71, 186.03424:143, 200.42559:72, 201.05589:395		√
34	Melibiose	5.1362	341.0513	2966.667	18189.72	–0.3	C12H22O11	O-glycosyl compounds	161.02791:89, 237.02626:63, 249.03312:63, 322.91631:71, 341.05064:382		√
35	Syringetin-3-O-glucoside	5.283283	507.1339	98991.78	486781.6	1.9	C23H24O13	Flavonoid-3-O-glycosides	182.02268:810, 181.01233:1270, 153.0227:250, 183.03011:2765, 387.09438:300, 345.08229:5361, 344.07561:783, 327.06945:408	√	√
36	Tartrate	5.6386	149.0231	6820.444	28992.52	5.2	C4H6O6	Sugar acids and derivatives	77.03966:143, 105.03373:71	√	√
37	Isorhamnetin-3-O-glucoside	5.663933	477.0673	8053	34217.96	–0.1	C22H22O12	Flavonoid-3-O-glycosides	299.99108:179, 314.99752:107, 315.01757:179	√	
38	Gamma-terpinene	5.674167	135.0449	24350.11	115035.9	0.4	C10H16	Branched unsaturated hydrocarbons	92.02841:179, 93.03642:143, 108.02875:108, 134.63319:146, 135.04317:708		√
39	Caffeic acid	5.674167	179.035	27090.45	131255.5	0.7	C9H8O4	Hydroxycinnamic acids	65.03737:107, 76.97366:107, 90.99894:109, 92.02705:184, 93.03642:307, 95.01522:107, 108.01994:107, 116.95741:179, 134.98574:187, 135.04481:4143, 178.97715:183, 179.03572:1028		√
40	Quercitrin	5.813	447.1505	3382.778	13813.96	–0.9	C21H20O11	Flavonoid-3-O-glycosides	149.04096:107, 243.0498:107, 379.15451:437, 401.12812:143, 447.09337:477		√
41	2-Methylglutaric acid	6.02025	145.0297	66289.78	277259.8	–0.9	C6H10O4	Methyl-branched fatty acids	101.04137:220, 145.02765:3466	√	
42	N-Acetyl-dl-Serine	6.036483	146.0243	2924.111	12888.43	4	C5H9NO4	N-acyl-L-alpha-amino acids	101.95179:36, 146.02907:214		√
43	Syringetin-3-O-galactoside	6.084567	507.1182	5577.667	26668.96	–6.7	C23H24O13	Flavonoid-3-O-glycosides	315.05017:107, 344.04437:143, 326.04115:107	√	√
44	Uridine 5′-monophosphate	6.2383	323.15	6317.333	22485.65	7.4	C9H13N2O9P	Pyrimidine ribonucleoside monophosphates	96.95784:412, 323.15244:1322		√
45	(±)-cis,trans-abscisic acid	6.345967	263.1308	5433.111	21122.29	–2.4	C15H20O4	Abscisic acids and derivatives	151.07204:214, 152.08225:399, 153.09058:286, 201.12978:214, 204.1191:286, 218.93645:144, 219.13711:849, 263.13184:322		√
46	L-Saccharopine	6.360717	275.0572	25859.78	132584.6	–0.3	C11H20N2O6	Glutamic acid and derivatives	233.0471:752, 189.0552:288, 83.01127:551, 135.04142:329, 147.04608:179, 174.0296:179, 163.04328:179	√	√
47	Xanthine	6.400383	151.0396	1941.667	24905.67	1.6	C5H4N4O2	Xanthines	65.00314:71, 108.01852:107	√	
48	Chlorogenic acid	6.47645	353.088	2378.778	13979.9	–2.7	C16H18O9	Quinic acids and derivatives	96.96051:71, 145.02707:71, 147.05461:143, 153.01195:71, 191.03696:143, 233.03596:71, 273.17521:71, 284.89935:71, 290.89474:71, 293.05695:71, 308.87808:71, 309.06923:71, 352.85347:71, 353.10023:143		√
49	Xanthosine-5′-monophosphate	6.47645	363.0856	1864.778	11378.01	–0.8	C10H13N4O9P	Purine ribonucleoside monophosphates	124.02087:71, 238.89106:71, 276.9039:71, 294.89482:71, 298.87338:71, 300.86653:107, 344.90829:71, 362.88796:71, 363.07631:286		√
50	1-O-*b*-d-glucopyranosyl sinapate	6.565617	385.1831	116762.4	448775.1	7.4	C17H22O10	Hydroxycinnamic acid glycosides	59.01515:487, 71.01369:418, 89.02662:495, 151.07385:408, 152.08232:1263, 153.09239:2611, 161.04594:758, 205.12723:858, 223.13367:915		√
51	Luteolin-6-C-glucoside	6.565883	447.0921	49076.55	412044.7	0.8	C21H20O11	Flavonoid C-glycosides	285.04563:250, 293.082:143, 297.03863:329, 299.05986:286, 300.01066:143, 311.04965:143, 327.05174:2158, 357.07186:828, 401.14331:143, 429.07598:143	√	√
52	Homogenentisic acid	6.7167	167.0358	13536.11	179889.9	–2.9	C8H8O4	2(hydroxyphenyl)acetic acids	125.0266:473	√	√
53	alpha-D-Galactose-1-phosphate	6.754533	259.0603	3629.556	33558.64	3.1	C6H13O9P	Monosaccharide phosphates	101.041:214, 129.02948:143, 146.95715:214, 157.02817:179, 213.02177:874	√	
54	Glucoerucin	6.838533	420.1306	42356.67	137832.1	–0.8	C12H23NO9S3	Alkylglucosinolates	257.07166:1174, 258.07348:444, 199.03863:549, 197.06307:146, 200.05009:143, 256.06953:109	√	
55	Sorbitol 6-phosphate	6.862433	261.0778	12360.44	46697.79	6.8	C6H15O9P	Monosaccharide phosphates	79.95471:250, 87.99967:79, 96.95931:2303, 174.96061:143, 196.91623:107, 260.98535:80, 261.0789:2793, 262.08392:143		√
56	Myricitrin	6.955433	463.0645	9139	39260.24	1.7	C21H20O12	Flavonoid-3-O-glycosides	419.07964:143, 435.06976:143, 463.06977:2532		√
57	Okanin-4′-O-glucoside	6.9811	449.0666	55377.33	202636.8	8.9	C21H22O11	Flavonoid O-glycosides	198.03323:1034, 199.04014:3086, 242.02041:1523, 405.07774:4668, 449.0662:2977		√
58	Apigenin 8-C-glucoside	7.02135	431.0973	338815.3	1509498	2.7	C21H20O10	Flavonoid 8-C-glycosides	117.03264:514, 135.0469:286, 149.02785:357, 159.04726:255, 161.02502:592, 163.03483:325, 191.03188:291, 203.03582:250, 239.07323:250, 243.07281:214, 268.04207:518, 269.03954:631, 281.04477:553, 282.04715:304, 283.0632:8569, 293.04846:559, 295.05609:324, 309.04316:250, 310.08679:339, 311.05744:29356, 323.04946:859, 341.0675:3446, 353.05654:361, 429.69683:220	√	√
59	Isoquercitrin	7.334	463.0865	19617.78	181288.4	–0.2	C21H20O12	Flavonoid-3-O-glycosides	299.99117:222, 300.02542:1037, 301.03656:879, 343.03668:107, 271.02818:143	√	
60	Taxifolin	7.334	303.0489	9169.667	72945.63	6.7	C15H12O7	Flavanonols	124.02737:109, 125.02338:544, 175.03928:143, 177.01607:143, 275.05138:143, 285.04576:557	√	
61	E-4,5′-dihydroxy-3-methoxy-3′-glucopyranosylstilbene	7.359167	419.0918	2477.667	10936.65	10	C21H24O9	Stilbene glycosides	146.97274:71, 214.94319:71, 256.03569:214, 257.04908:71, 282.93447:71, 400.88298:71, 418.90958:72	√	√
62	E-3,4,5′-Trihydroxy-3′-glucopyranosylstilbene	7.447834	405.0811	30828.67	196134	–0.5	C20H22O9	Stilbene glycosides	243.02839:3249, 242.02547:991, 186.0286:509, 187.04124:377, 215.03425:214, 159.03972:179, 241.99911:119	√	√
63	Kaempferol-7-neohesperidoside	7.548167	593.1527	97225.66	1592578	–0.9	C27H30O15	Flavonoid-7-O-glycosides	284.02392:348, 285.04113:7073, 591.22485:179, 269.04874:143, 327.04194:107, 447.10133:89, 547.10886:89, 133.02732:71	√	√
64	Catechin	7.5839	289.0739	3604	17984.94	–7.1	C15H14O6	Catechins	93.035:143, 112.9885:143, 158.97546:179, 174.9642:143, 201.09383:179, 245.08418:286, 289.07676:330		√
65	Quercetin-4′-glucoside	7.585667	463.0869	14341.67	139766.3	0.7	C21H20O12	Flavonoid O-glycosides	300.03532:361, 301.03913:1039, 271.03061:107, 299.99618:108, 461.62247:107	√	√
66	trans-Cinnamate	7.634233	146.961	12460.44	130252.1	0.4	C9H8O2	Cinnamic acids	102.96949:143, 147.0239:71		√
67	1-Myristoyl-2-hydroxy-*sn*-glycero-3-phosphate	7.807817	381.1565	3523.444	15262.15	6.4	C17H35O7P	1-acylglycerol-3-phosphates	96.95781:143, 380.97838:71	√	√
68	Maritimetin-6-O-glucoside	7.84615	447.0925	259140.3	2822031	1	C21H20O11	Aurone O-glycosides	133.0288:143, 151.00277:143, 199.04059:179, 211.03944:143, 256.03572:217, 284.03312:4322, 285.04081:23021, 286.04551:323, 300.00086:143, 327.05945:483, 357.05596:179, 445.66263:181	√	√
69	Eriodictyol-7-O-glucoside	8.146633	449.0714	68401.22	288372.2	6	C21H22O11	Flavonoid-7-O-glycosides	133.02869:179, 151.00267:214, 152.00569:143, 198.03362:328, 199.03853:179, 242.02104:600, 284.03074:250, 285.0432:3654, 286.04552:3016, 287.0472:6715, 448.11391:114	√	√
70	Pelargonidin-3-O-glucoside	8.183967	431.1004	445132.4	2154139	–2.8	C21H21O10	Anthocyanidin-3-O-glycosides	267.03488:253, 268.03511:13883, 269.04415:41490, 283.06081:328, 311.05747:1214	√	
71	Unknown	8.27145	417.1195	6675.556	24871.43	–1.2	C20H18O10		144.94608:145, 145.03621:401, 212.93365:143, 280.924:214, 307.07745:179, 348.9131:107	√	
72	Raffinose	8.283783	503.1795	3161.667	11441.96	–4.3	C18H32O16	Oligosaccharides	323.0164:73, 485.01493:73	√	
73	Daidzein-8-C-glucoside	8.333616	415.1949	30877.89	113332.9	6.8	C21H20O9	Isoflavonoid C-glycosides	59.01294:107, 89.02401:71, 119.02697:71, 161.04805:71, 179.05875:286, 278.94602:71, 378.84498:71	√	√
74	Apigenin-7-O-glucoside	8.346117	431.1	593328.6	3172254	–1.8	C21H20O10	Flavonoid	117.03544:357, 157.06376:73, 159.05055:71, 161.02295:71, 201.05822:322, 211.03958:442, 225.05599:214, 227.03492:109, 229.01397:73, 239.03803:556, 240.0413:1099, 241.04668:71, 253.04361:214, 268.03717:27419, 269.04159:21766, 271.67023:71, 283.06768:625, 311.0522:1405, 337.05469:73, 341.06464:474, 413.07991:71, 429.70539:754	√	√
75	Eriodictyol-7-O-neohesperidoside	8.446116	595.1146	2948.111	16343.76	–1	C27H32O15	Flavonoid-7-O-glycosides	109.02641:73, 284.0454:74, 286.03156:71, 285.03881:589, 287.04758:107, 594.14713:107, 447.08933:364	√	
76	Phlorizin	8.661933	435.168	27466.45	211998.5	–1.1	C21H24O10	Flavonoid O-glycosides	273.11537:143, 327.17443:76	√	√
77	3′-Methoxy-4′,5,7-trihydroxyflavonol	8.687433	315.0506	79890.66	391934.1	1.3	C16H12O7	Flavonols	227.07097:1009, 246.91872:325, 271.05825:479, 299.99837:804, 300.00326:571, 185.05905:401	√	√
78	Naringenin	8.846666	271.022	4319.889	22277.4	5.1	C15H12O5	Flavanones	116.95142:60, 171.05047:71, 225.01321:60, 243.02362:95, 270.99732:85, 271.02522:866		√
79	Rhoifolin	8.940084	577.195	1787	7811.248	–9.4	C27H30O14	Flavonoid	145.0238:71, 163.03256:54, 531.01855:71	√	√
80	4-deoxyphloridzin	8.940084	419.1036	7305	57402.61	–7.2	C21H24O9	Flavonoid	173.05668:71, 229.05227:71, 257.04453:603	√	√
81	3-Hydroxy-3-Methylglutaric acid	9.129084	161.0596	8163.222	39133.83	6.2	C6H10O5	Hydroxy fatty acids	72.99451:107, 73.00054:107, 98.94957:71, 116.99564:220, 117.03384:516, 118.04591:179, 119.04539:582, 145.02731:107, 146.02909:71, 160.98345:74	√	√
82	Kaempferol-3-O-alpha-L-rhamnoside	9.166417	431.097	16025.78	95502.76	2	C21H20O10	Flavonoid-3-O-glycosides	284.03558:250, 285.04327:2832, 430.85368:74	√	√
83	3′,4′,5,7-tetrahydroxyflavanone	9.654716	287.0564	34029.78	419093.6	–0.8	C15H12O6	Flavanones	151.00297:3269	√	√
84	Quercetin	10.2566	301.0355	8108.778	81530.34	0.9	C15H10O7	Flavonols	150.99591:107, 151.00285:179, 179.00563:125, 214.91186:107, 232.92412:253, 300.99908:148, 301.03828:776		√
85	Luteolin	10.55558	285.0422	186724	3137542	–6	C15H10O6	Flavones	107.01414:112, 132.02269:128, 133.02906:1360, 149.02759:133, 150.99775:200, 151.00469:466, 175.03732:313, 199.04056:260, 201.01797:123, 241.04631:143, 285.04032:7041, 286.04024:396		√
86	UDP-beta-L-rhamnose	10.88123	549.1422	1174.556	5956.012	–2.7	C15H24N2O16P2	Pyrimidine nucleotide sugars	363.11171:143, 375.10674:143, 504.82505:72, 549.13686:518		√
87	Apigenin	11.04865	269.0449	541693.4	6306418	2.1	C15H10O5	Flavones	63.0232:786, 64.99937:312, 65.00279:1425, 83.0137:622, 93.03332:204, 105.03299:394, 107.0121:1756, 117.03347:9816, 121.02865:777, 149.02368:4137, 150.99903:2595, 151.0025:5105, 157.06153:214, 159.04653:1577, 161.0207:335, 169.06486:416, 180.0556:499, 181.06708:1002, 182.03757:262, 183.04504:1029, 185.02222:250, 196.05095:262, 197.06273:460, 201.05576:1340, 224.04606:368, 225.05553:2814, 227.03234:1426, 241.04622:288, 268.18242:510	√	√
88	Acacetin	11.18413	283.022	14729.56	72032.9	9.1	C16H12O5	4′-O-methylated flavonoids	146.96075:433, 165.03325:144, 193.03174:214, 195.04424:1600, 214.94952:143, 221.02966:179, 239.02498:325, 265.0054:286	√	√
89	3 5 7-Trihydroxy-4′-methoxyflavone	11.35647	299.0573	16476.67	167306.8	0.3	C16H12O6	Flavonols	284.03086:1686, 256.03814:250, 199.03883:89, 162.96251:161, 163.9556:54, 227.02647:71, 230.93942:54, 255.0696:54, 271.02354:54, 151.0013:71	√	√
90	2′-Deoxyinosine 5′-monophosphate	13.06028	331.0658	4829.556	27894.9	–9.3	C10H13N4O7P	Purine 2′-deoxyribonucleoside monophosphates	176.92754:86, 331.06281:806		√
91	Prostaglandin E1	15.67557	353.198	8006.778	40175.36	6.9	C20H34O5	Prostaglandins and related compounds	95.94846:10, 96.96097:150, 122.97461:21, 285.04107:133, 290.87414:10, 308.88418:10, 334.8752:10, 335.00444:12, 352.08053:17	√	√
92	Esculin	16.71335	339.2001	45158	242293.7	0.8	C15H16O9	Coumarin glycosides	119.04833:125, 182.99939:371, 183.01085:1357, 292.91251:250, 293.21714:161, 338.10526:107	√	√
93	5-Aminoimidazole-4-carboxamide-1-ribofuranosyl 5′-monophosphate	16.9815	337.2029	4037.444	19757.09	6.9	C9H15N4O8P	1-ribosyl-imidazolecarboxamides	96.96054:131	√	√
94	Gamma-Linolenic acid	20.51148	277.2171	597667	8168999	1	C18H30O2	Lineolic acids and derivatives	59.01437:155, 127.07398:107, 208.96073:119, 233.22685:264, 259.20068:313, 275.20167:226, 276.32269:363	√	√

**Table 4:** Metabolites Tentatively Identified Using Q-TOF LC/MS/MS in the Alcoholic (HC) and Aqueous Extracts (HQ) of the Aerial Parts of LI in Positive Ionization Mode

										*L. inermis*	
No.	Identification	RT (Min.)	Precursor (*m*/*z*) M+	Height	Area	Error (PPM)	Formula	Chemical class	Fragmentation (*m*/*z*:int.)	HC	HQ
1	S-Lactoylglutathione	1.156617	380.0965	141657.6	646132.1	–1.7	C13H21N3O8S	Oligopeptides	104.10997:143, 218.03897:215, 260.0407:580, 296.07368:773	√	√
2	Daidzein	1.156617	255.1347	15199.22	83490.52	1.3	C15H10O4	Isoflavones	140.96577:72	√	
3	Uracil	1.183283	112.8949	8591.667	30865.05	0.8	C4H4N2O2	Pyrimidones	68.98582:72	√	
4	l-Arginine	1.2185	175.1183	24815.78	208939.9	–0.5	C6H14N4O2	L-alpha-amino acids	60.05737:328, 70.06696:1642, 72.0861:143, 116.07555:322, 130.09834:179, 158.09313:216, 175.12333:617		√
5	L-tryptophan	1.232	205.0666	529026.1	3397475	6.9	C11H12N2O2	Indolyl carboxylic acids and derivatives	87.04163:215, 145.01241:107, 149.02935:179, 204.43032:215, 205.0729:8225		√
6	N,N-Dimethylglycine	1.23595	104.106	585297.4	3737162	2.6	C4H9NO2	Alpha amino acids	51.026:73, 55.38852:73, 56.04839:697, 57.89446:347, 58.06651:25138, 59.07593:2404, 60.0831:14784, 69.03469:143, 71.07472:143, 86.09223:143, 87.04349:179, 88.08214:143, 103.78725:179	√	
7	Sorbitol	1.23595	183.0842	176711.2	1162196	8.5	C6H14O6	Sugar alcohols	55.05481:918, 57.03592:1064, 61.03036:1246, 69.03469:7770, 73.03096:518, 81.03757:441, 83.05086:1398, 85.03156:1563, 87.04876:634, 99.04617:675, 111.04864:903, 129.05248:659, 147.06597:769	√	√
8	Choline	1.245	104.1069	584993.9	3773231	–1.9	C5H14NO	Cholines	56.04904:703, 57.88653:322, 58.06502:24373, 59.07443:2407, 60.0794:18118, 103.78822:322, 104.10786:10644		√
9	1,4-Benzoquinone	1.258017	109.0278	4665.333	54370.38	0.3	C6H4O2	P-benzoquinones	53.03504:72, 55.01541:72, 67.05493:72, 77.04101:107, 81.03306:72, 109.08194:72		√
10	Xanthosine-5′-monophosphate	1.26145	365.1024	148330	1235082	7.1	C10H13N4O9P	Purine ribonucleoside monophosphates	185.04378:612, 203.05834:1904, 347.09736:701	√	
11	Trigonelline	1.274783	138.0548	505180.3	2637560	–0.7	C7H7NO2	Alkaloids and derivatives	50.01443:1454, 51.02215:2541, 52.03177:1498, 53.041:2500, 65.03941:9121, 66.0363:1555, 67.05464:1920, 78.03297:9119, 79.04166:3989, 80.04925:1271, 92.04909:15081, 93.05987:4035, 94.06795:13745, 136.04318:1233	√	√
12	4-Methyl-5-thiazoleethanol	1.327283	144.1011	31171.45	138636.6	0.9	C6H9NOS	4,5-disubstituted thiazoles	55.05514:143, 58.06469:1211, 68.05063:107, 70.06198:107, 72.08103:107, 84.08137:486, 143.6713:113	√	√
13	Aniline	1.3406	94.06473	5765.667	44263.41	–3.7	C6H7N	Aniline and substituted anilines	50.01458:250, 51.02432:250, 52.02988:435, 65.03956:107, 67.05247:72, 77.0309:72, 78.03061:286, 79.0418:569, 80.04685:72, 92.04921:72, 93.05726:110	√	
14	Indoleacetic acid	1.3406	176.0904	14543.56	64404.27	6	C10H9NO2	Indole-3-acetic acid derivatives	55.05724:251, 71.04887:258, 87.04121:404, 98.06238:215, 112.07519:215, 117.05686:358, 130.09124:578	√	
15	Phenylalanine	1.353433	166.084	9744.333	43370.19	4.6	C9H11NO2	Phenylalanine and derivatives	59.04805:107, 73.06751:143, 80.04685:215, 87.04648:107, 107.07029:107	√	
16	Okanin-4′-O-glucoside	1.353433	451.1337	3325.556	26364.05	1.3	C21H22O11	Flavonoid O-glycosides	273.07558:107, 289.11219:143, 405.07952:143, 433.12979:143, 451.14702:953	√	
17	Cytidine	1.364333	244.082	10948.56	78325.06	1.2	C9H13N3O5	Pyrimidine nucleosides	112.05189:502, 244.07665:688		√
18	Tyrosine	1.367433	182.0806	34681.33	166773.7	3.3	C9H11NO3	Tyrosine and derivatives	78.04051:364, 87.04377:561, 92.05184:560, 94.06799:557, 96.03955:1132, 136.07286:511, 138.06012:2238, 152.03629:1080, 167.06564:1052	√	√
19	Phenylephrine	1.367433	168.0643	13933.33	63217.91	5.3	C9H13NO2	1-hydroxy-4-unsubstituted benzenoids	68.05284:107, 96.04503:718, 108.04849:143, 122.05935:179, 124.04881:107, 150.07493:143	√	√
20	L-beta-Homoleucine	1.367433	146.0911	92105.55	358331.6	7.3	C7H15NO2	Beta amino acids and derivatives	56.04863:358, 60.05708:857, 69.0373:993, 83.06143:394, 86.06368:3491, 87.0464:4582, 104.07547:555, 111.06084:468, 128.08564:466	√	
21	4-Aminophenol	1.367433	110.0574	9026.777	57583.75	9.7	C6H7NO	Aniline and substituted anilines	66.01823:24, 67.04106:72, 68.98123:143, 77.04592:24, 80.04953:36, 82.07361:24, 92.06562:24, 95.04042:24, 110.06322:131	√	√
22	Melatonin	1.378333	233.0635	48442	670803.4	0.3	C13H16N2O2	3-alkylindoles	84.95685:362, 128.07361:143, 158.98934:107, 159.00358:143, 201.04026:107, 233.06234:1705		√
23	Glycerophosphate	1.419667	172.9759	2536.556	21302.32	0	C3H9O6P	Glycerophosphates	70.06472:107, 98.97682:72, 173.04403:72		√
24	3 3′ 4′ 5-tetrahydroxy-7-methoxyflavone	1.421933	317.0794	12607.56	124522.9	9.5	C16H12O7	Flavonols	166.96325:72, 196.95918:72, 240.95234:72, 252.88094:72, 253.01569:72, 281.0248:72, 299.08952:73	√	√
25	L-beta-Homoproline	1.4351	130.0477	32218.67	216428.9	9.2	C6H11NO2	Pyrrolidines	55.05505:144, 56.04863:433, 68.05287:179, 69.84928:289, 70.06662:15097, 71.04878:322, 84.08388:1042, 112.0871:517	√	√
26	N1-Acetylspermine	1.4475	245.0626	36183.11	405998.5	2.5	C12H28N4O	Acetamides	84.95944:107, 100.11241:72, 105.06964:72, 113.04066:143, 120.95805:72, 138.96506:72, 198.93145:72, 203.05968:72, 204.05498:72, 244.13852:74, 245.07055:1528		√
27	Adenine	1.680433	136.0627	7644	82821.57	–9.4	C5H5N5	6-aminopurines	92.0248:179, 109.01662:216, 119.03914:508, 136.05966:840	√	√
28	Pipecolate	1.706083	130.0958	5012.333	40313.68	3.5	C6H11NO2	Alpha amino acids	56.05069:358, 70.06419:358, 77.03819:107, 84.04497:1105, 103.05504:143	√	
29	Resveratrol	1.835817	229.1543	3920.444	23021.26	0.8	C14H12O3	Stilbenes	60.08144:107, 70.06695:251, 99.01323:107, 142.08532:215, 198.10763:73, 229.16373:661		√
30	Nicotinamide	2.071233	123.0538	12213.67	101912.5	0.6	C6H6N2O	Nicotinamides	51.02416:107, 53.04099:251, 78.03795:358 ,79.05421:147, 80.04924:639, 81.07592:107	√	√
31	Pyridoxine	2.071233	170.0788	4710.222	74079.77	7.7	C8H11NO3	Pyridoxines	79.05918:107, 123.05673:73, 134.06392:540, 152.07448:179	√	√
32	Piperidine	2.292717	86.05934	11683	188665.8	0.9	C5H11N	Alkaloids	69.03256:179, 85.80444:73	√	√
33	Hesperetin	4.871417	303.1398	7805	55376.05	1.6	C16H14O6	4′-O-methylated flavonoids	84.96161:54, 90.9739:54, 214.91426:54, 234.95779:54, 238.90954:72, 256.92593:125, 284.88271:72, 302.89208:54	√	
34	Luteolin-8-C-glucoside	4.932317	449.1198	1298.889	13931.01	2.9	C21H20O11	Flavonoid 8-C-glycosides	214.92797:36, 266.92364:24, 310.83448:24, 312.92438:24, 316.87374:24, 319.09479:24, 330.84016:24, 356.84328:24, 366.86291:24, 384.85989:48, 402.79449:24, 403.06662:24, 412.84409:24, 430.8197:24, 431.107:24, 449.13048:155		√
35	Daphnetin	5.005417	179.0336	7053.556	69381.84	–0.7	C9H6O4	7,8-dihydroxycoumarins	51.02231:215, 74.99701:73, 77.03836:253, 84.96164:107, 105.03438:215, 119.08856:73, 132.61937:73, 133.03276:877	√	
36	4-Methoxycinnamic acid	5.025983	179.0344	3419.667	34468.82	–0.2	C10H10O3	Cinnamic acids	51.02252:119, 77.03624:215, 79.05475:60, 105.04069:107, 133.03344:621, 179.03583:72		√
37	Naringenin	5.032084	273.0922	39047.11	286300.9	6.8	C15H12O5	Flavanones	138.96786:107, 166.97467:72, 173.0879:72, 190.94181:72, 198.93454:143, 213.05531:72, 229.08149:73, 236.94063:107, 255.18616:111, 272.97794:75	√	
38	Kynurenic acid	5.102033	190.0491	8291.556	67908.55	–0.6	C10H7NO3	Quinoline carboxylic acids	84.9616:215, 89.04199:401, 116.05066:438, 144.04735:1796, 162.05869:215, 172.0451:179	√	
39	Thymine	5.137583	127.0412	3933.111	45005.12	2.8	C5H6N2O2	Hydroxypyrimidines	71.9516:72	√	
40	Galactinol Dihydrate	5.43295	343.1361	9551.777	61661.2	0.5	C12H22O11	O-glycosyl compounds	117.09422:72, 163.03545:72, 232.89862:72, 250.91029:125, 268.93:125, 342.06615:72, 343.14829:1562		√
41	3-Formylindole	5.500383	146.059	3629.667	36755.83	1.2	C9H7NO	Indoles	77.03855:143, 90.04176:72, 91.05227:143, 117.05409:143, 118.06771:72, 128.05082:108	√	
42	Jasmonic acid	5.831533	210.9965	13434.67	59861.39	1.6	C12H18O3	Jasmonic acids	62.98331:179, 95.08985:72, 137.02344:72, 193.17265:72	√	
43	7-Acetoxy-4-methylcoumarin	6.0412	219.099	3504.556	21661.08	–0.6	C12H10O4	Coumarins and derivatives	84.95914:179, 203.14325:107	√	
44	Phosphoenolpyruvate	6.080033	169.0491	52550.89	352815.6	0.6	C3H5O6P	Phosphate esters	55.01952:215, 67.0201:633, 68.99985:724, 95.0513:618, 123.04753:1422, 127.04261:286, 151.04199:1151	√	
45	12-Oxo-10,15(Z)-Phytodienoic Acid	6.31335	293.0995	38588.78	255677.6	0.9	C18H28O3	Prostaglandins and related compounds	206.90793:72, 260.76995:75, 292.19381:76	√	√
46	6,7-Dihydroxycoumarin	6.422567	179.0344	4487.667	32120.25	–0.3	C9H6O4	6,7-dihydroxycoumarins	51.02269:108, 77.0389:144, 105.03798:72, 119.09242:73, 123.04523:470, 133.03036:395, 151.03952:108, 179.03598:287		√
47	Quercetin	6.578166	303.0463	9101.111	72056.3	–0.3	C15H10O7	Flavonols	131.0562:72, 171.09798:72, 214.91831:72, 226.09862:72, 228.9444:72, 234.95769:72, 238.93126:107, 241.04893:72, 256.93034:72, 284.85875:72, 285.13529:72, 302.88211:72	√	
48	Sabinene	6.57995	137.0588	4591.111	29462.03	–0.3	C10H16	Bicyclic monoterpenoids	51.02265:50, 65.04001:79, 66.04379:100, 81.07409:57, 94.04408:201, 122.03851:129, 137.06405:222		√
49	Sissotrin	6.603833	447.1859	8111.333	51236.65	0.8	C22H22O10	Isoflavonoid O-glycosides	283.00285:54, 354.87521:54, 382.85451:54, 445.7822:72	√	
50	L-beta-Homothreonine	6.68205	134.0583	24665.33	168078.1	3.8	C5H11NO3	Beta amino acids and derivatives	89.04002:1037, 116.05151:2868, 134.06177:2976		√
51	Procyanidin B2	6.995983	579.1757	3854.333	25437.92	9.5	C30H26O12	Biflavonoids and polyflavonoids	401.17283:72, 533.15598:107, 561.15199:72, 563.22795:107	√	√
52	4′-Hydroxyisoflavone-7-O-glucoside	7.08615	417.1735	73835.34	517691	–1.8	C21H20O9	Isoflavonoid O-glycosides	285.13551:287, 415.87164:125	√	√
53	Pyridoxamine	7.2132	169.05	1119.222	9926.002	–3.7	C8H12N2O2	Pyridoxamine 5′-phosphates	67.05293:72, 68.98154:72, 91.05533:72, 95.08771:72, 169.0328:72		√
54	Chalcone	7.2163	209.1178	3408.444	22781.12	0.6	C15H12O	Retrochalcones	75.00446:72, 77.04591:72, 78.99926:72, 79.05952:72, 83.04622:72, 91.05219:72, 93.06832:72, 105.07213:72, 107.08215:72, 117.00511:72, 119.08868:107, 131.08552:72, 135.11278:72, 149.09082:72	√	
55	Kaempferol-3-O-rutinoside	7.410133	595.1657	109317.6	604421.4	0.1	C27H30O15	Flavonoid-3-O-glycosides	57.03635:144, 71.05136:396, 85.03203:363, 129.05298:430, 147.06989:143, 153.01663:179, 286.16182:251, 287.06074:23010, 433.13017:72, 447.67473:107, 449.11606:8688, 577.16782:72	√	√
56	Hinokitiol	7.478183	165.1276	1881.111	12696.33	–3.7	C10H12O2	Tropolones	53.0374:36, 57.03239:36, 60.99143:36, 65.03774:36, 67.05295:36, 69.06839:36, 77.03888:36, 78.99968:36, 79.05492:36, 81.07156:72, 82.99777:36, 90.97724:36, 91.05538:36, 93.04431:36, 95.01067:36, 101.05016:36, 107.0769:36, 109.06168:36, 119.04621:36, 120.94272:36, 123.08912:36, 124.03096:36, 129.07943:36, 136.08047:36, 147.1256:36, 165.13764:72		√
57	Benzoic acid	7.53085	123.1158	2967.333	28228.43	3.4	C7H6O2	Benzoic acids	55.05358:107, 60.98937:72, 79.05757:72, 81.07932:72, 123.09235:143		√
58	Hyperoside	7.611117	465.1002	7113.556	68994.25	3.9	C21H20O12	Flavonoid-3-O-glycosides	153.02708:107, 257.05283:73, 292.8946:75,303.05938:2402, 317.07823:107	√	√
59	Sinapyl aldehyde	7.8501	209.0199	42057.89	213582.3	–4.3	C11H12O4	Methoxyphenols	62.9856:107, 93.02464:72, 120.95437:72, 128.94387:72, 135.02407:72, 149.02522:72, 165.03136:107, 191.16032:73, 193.01182:73, 208.99756:73	√	√
60	Genistein	7.886766	270.9895	28699.67	278412	–0.2	C15H10O5	Isoflavones	124.95676:143, 206.92053:107, 224.90299:107	√	√
61	Ononin	7.973267	431.1677	4328.333	29728.53	0.4	C22H22O9	Isoflavonoid O-glycosides	268.01691:143, 269.04437:72	√	√
62	Cyanidin-3, 5-di-O-glucoside	8.128917	611.1605	1456.333	13028.91	–9.4	C27H31O16	Anthocyanidin-5-O-glycosides	287.06069:474, 355.09353:107, 356.07348:143, 565.16012:107	√	
63	Adenosine	8.312917	268.1098	44836.78	290353	–3.2	C10H13N5O4	Purine nucleosides	121.07857:215, 153.07241:215, 181.06995:215, 197.05854:251, 198.05494:648, 211.06859:269, 226.09025:2994, 240.08096:931, 250.09978:215, 251.08339:269	√	√
64	Gossypin	8.3248	481.1726	3501.222	24383.5	–2.4	C21H20O13	Flavonoid-8-O-glycosides	317.09518:54, 481.17872:1142		√
65	Cyanidin-3-glucoside	8.391067	449.1083	504173.4	3340514	0.5	C21H21O11	Anthocyanidin-3-O-glycosides	135.04387:578, 137.03024:215, 153.0202:1944, 161.02149:287, 241.05802:215, 269.04447:435, 271.07274:292, 286.16193:1323, 287.06084:91641, 447.68681:215	√	√
66	Peonidine-3-O-glucoside chloride	8.6009	463.132	6943.111	79215.34	0.7	C22H23O11	Anthocyanidin-3-O-glycosides	286.0425:540, 301.08123:2585, 395.21025:1353, 463.15238:515	√	
67	Riboflavin	8.7034	377.1209	61122.55	418100.9	–0.8	C17H20N4O6	Flavins	187.0436:179, 213.07593:107, 222.89552:72, 289.07416:72, 290.88253:72, 312.92278:72, 375.9507:107	√	
68	4-Hydroxy-3-methoxycinnamaldehyde	8.72045	179.1062	8756.777	40895.29	1.2	C10H10O3	Methoxyphenols	53.04146:107, 77.04127:107, 91.05527:287, 105.06969:215, 115.05552:107, 117.07284:107, 133.10189:287, 161.09387:107, 179.11141:322		√
69	L-beta-homotryptophan-HCl	8.729234	219.0992	42312.45	320910.7	–0.6	C12H14N2O2	Beta amino acids and derivatives	84.95647:107, 200.91519:143, 218.40645:109	√	√
70	3,4-Dimethoxycinnamic acid	8.80405	209.1157	9247.556	73599.43	0.5	C11H12O4	Coumaric acids and derivatives	83.01267:72, 84.95386:72, 87.00439:72, 93.01374:72, 128.94386:72, 135.02405:72, 149.0252:107, 191.10562:72	√	
71	Coumaric acid	8.94655	165.1283	4017.667	17264.9	–3.3	C9H8O3	Hydroxycinnamic acids	67.06176:72, 79.05189:72, 81.07102:215, 91.05478:72, 105.07492:72, 109.06685:72, 120.95745:72, 123.08517:215, 147.04584:72	√	√
72	3-(4-Hydroxy-3,5-dimethoxyphenyl)-2-propenoic acid	9.0377	225.1478	4171.444	19075.79	1	C11H12O5	Hydroxycinnamic acids	84.96705:144, 85.02954:364, 123.12299:143, 207.13594:251	√	
73	2′-Deoxyadenosine	9.479183	252.0607	8054.444	53364.46	8.2	C10H13N5O3	Purine 2′-deoxyribonucleosides	57.07039:18, 84.04513:18, 121.10656:18, 138.0967:18, 158.99919:18, 180.92936:27, 217.0727:18, 235.2133:18, 251.28031:27	√	
74	S-Adenosyl-l-methionine	9.5379	399.1214	24860.45	157060.9	–0.6	C15H22N6O5S	5′-deoxy-5′-thionucleosides	234.91156:54, 353.06006:72, 399.1355:894		√
75	Unknown	9.622017	481.1825	10594.22	72341.16	–7.6	C21H20O13		90.97392:72, 130.99809:72, 228.9702:322, 291.16183:287, 307.15396:73, 309.18676:1509, 319.09808:72, 383.2195:143, 401.25767:143, 462.91518:72, 463.18867:179	√	
76	D-(+)-Trehalose	9.777	343.0114	9660.111	77381.79	–1.3	C12H22O11	O-glycosyl compounds	241.04906:322, 250.97151:394, 268.97482:143, 296.99804:288, 297.00291:326, 343.02609:434, 342.98425:179	√	√
77	Cyanidin-3-O-rutinoside	9.789333	595.142	4337.556	28185.52	1	C27H31O15	Anthocyanidin-3-O-glycosides	147.04575:394, 287.05591:1832, 309.09242:72, 456.98533:72	√	
78	S-Adenosyl-l-homocysteine	9.8945	385.1571	3328	25068.98	9	C14H20N6O5S	5′-deoxy-5′-thionucleosides	252.89507:72, 270.91488:72, 320.86668:107, 337.06831:107, 338.9064:179, 366.88288:72, 367.06684:107, 383.90571:73	√	√
79	Luteolin	10.20698	287.0559	813384.6	3572603	–2.4	C15H10O6	Flavones	68.9999:2348, 69.00224:1180, 89.03941:1322, 117.03254:1508, 135.04379:4977, 137.02354:1741, 153.02362:13461, 161.02858:2826, 213.05526:1166, 241.05361:2385, 269.0491:1664, 286.15716:1060	√	
80	Kaempferol-3-O-(6-p-coumaroyl)-glucoside	10.20698	595.0866	2526.778	10369.84	6.5	C30H26O13	Flavonoid 3-O-p-coumaroyl glycosides	287.05129:72, 309.033:215, 594.90346:109	√	
81	Malvidin-3-galactoside	10.6708	493.1166	6430.333	44885.05	–4.7	C23H25O12	Anthocyanidin-3-O-glycosides	285.05918:161, 313.06255:90, 331.06441:1638	√	
82	Cinnamaldehyde	10.85117	133.1018	3946.222	21452.98	–3.4	C9H8O	Cinnamaldehydes	73.06553:50, 77.03631:50,79.05482:50, 91.05527:64, 105.07549:50, 115.05856:72, 117.07286:57, 133.10518:115		√
83	Isopentenyladenine	13.79533	204.1385	12318.22	61927.82	0.6	C10H13N5	6-alkylaminopurines	116.05074:494, 134.06092:1290	√	√
84	Guanosine	13.86518	284.0674	2214.222	11001.28	2.7	C10H13N5O5	Purine nucleosides	100.11798:14, 178.93749:21, 182.96082:21, 206.93352:14, 219.94619:14, 226.12964:14, 242.94396:29, 247.89056:21, 266.18112:14, 284.07919:229		√
85	Cytidine-5′-diphosphate	14.1486	404.2102	22350.11	102158.6	–6.7	C9H15N3O11P2	Pyrimidine ribonucleoside diphosphates	105.06911:2472, 121.06618:1668, 267.12527:561, 387.18853:1300	√	√
86	1-O-*b*-d-glucopyranosyl sinapate	14.1486	387.1773	39836.89	189423.6	8.1	C17H22O10	Hydroxycinnamic acid glycosides	69.03504:155, 77.03835:96, 93.06819:96, 105.072:3219, 119.05465:96, 121.06618:382	√	
87	Isoguvacine	24.16948	128.1429	50134.78	578726.8	0.6	C6H9NO2	Hydropyridines	55.0537:470, 56.05361:366, 58.06752:768, 84.08746:143, 98.09697:488, 100.11574:143, 128.14771:6242		√

### 3.4 Q-TOF LC/MS/MS-Based Multivariate Data Analyses and Fingerprinting of Henna Extracts

Heterogeneity of the metabolic profile of both HC and HQ extracts was scrutinized by conducting multivariate data analyses in an untargeted manner. These separations between the extracts can be determined via visual inspection of the Q-TOF LC/MS/MS chromatograms, but executing the PCA will give more in-depth qualitative and quantitative information about the similarities and differences between both extracts, as well as their major marker and fingerprinting compounds; especially that there is a large number of characterized compounds in both of them.

**Figure 7:**
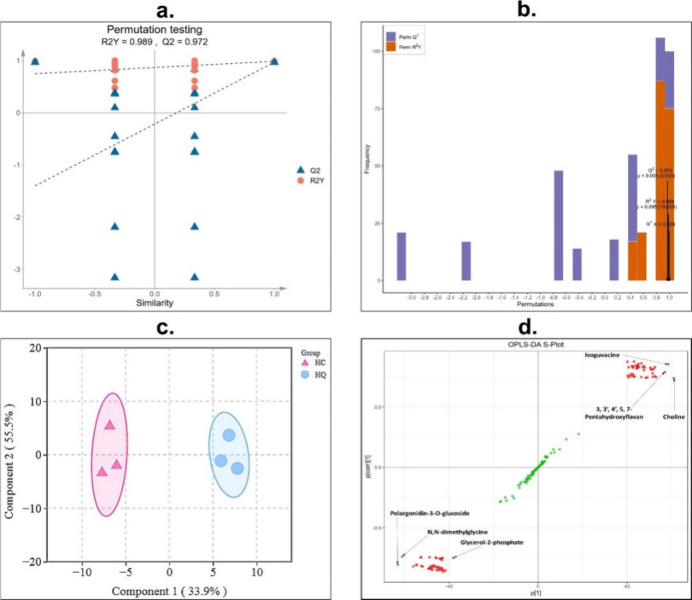
PCA and OPLS-DA modeling of metabolites in HC and HQ as analyzed by Q-TOF LC/MS/MS (*n* = 2). (a) OPLS-DA permutation test plot, (b) OPLS-DA model validation plot, (c) OPLS-DA score plot of PC1 vs PC2 scores, (d) OPLS-DA S-plot for the alcoholic versus aqueous extract of Henna, showing covariance p [1] against the correlation p (corr) [1] for the variables of the discriminating components of the OPLS-DA model.

The permutation testing and model validation plots (Figure 7a,b) indicated that values of R^2^Y, R^2^X, and Q^2^ are 0.989, 0.999, and 0.972, with *P* < 0.005, which indicates that the model is optimal and in a high degree of stability and reliability. The PCA of the Q-TOF LC/MS/MS dataset of the extracts was displayed by two orthogonal PCs as shown in the PCA score plot (Figure 7c), accounting for 33.9% of the total variance prescribed by PC1 that indicates the amount of separation between the tested extracts/groups, and 55.5% for PC2 which reflects the quantity of the intragroup sample variation. HC positioned toward the right side (positive score values) along PC1, whereas HQ clustered in the left side of PC1 (negative score values).

The illustrative OPLS-DA S-plot in Figure 7d demonstrated some overlap between HC and HQ, but a clear segregation between both extracts is lucid, showing 45 phytochemicals as markers for HQ, while 40 compounds for HC. The major marker compounds in HQ are isoguvacine, 3,3′,4′,5,7-pentahydroxyflavan, and choline, while the major ones in HC are glycerol-2-phosphate, *N*,*N*-dimethylglycine, and pelargonidin-3-O-glucoside.

### 3.5 Evaluation of Antioxidant Activity (*In Vitro*)

The *in vitro* ABTS and DPPH radical scavenging assays are commonly used for the evaluation of the radical scavenging abilities or antioxidant effects of natural products’ extracts and their derived NPs against ABTS and DPPH radicals. These radicals are foreign and harmful to our biological system. Both are spectrophotometric techniques based on quenching of the stable colored ABTS·+ and DPPH radicals.^57^

**Figure 8:**
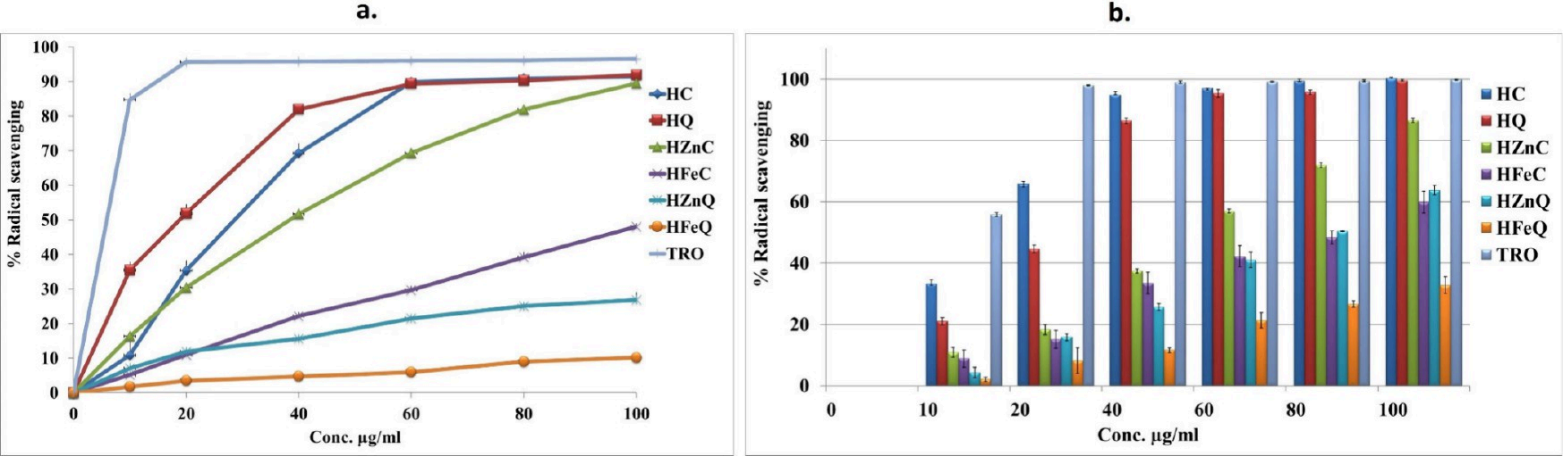
In vitro antioxidant activities: (a) DPPH and (b) ABTS assays of the utilized plant extracts and their corresponding biofabricated NPs. Data are illustrated as means ± SE (*n* = 3).

Both the ABTS and DPPH assays measure antioxidant activity based on the reduction of unstable radicals. In the ABTS assay, a blue-green radical (ABTS·+) is reduced to a colorless form by the antioxidants. Similarly, the DPPH assay measures the reduction of the purple DPPH· radical to its colorless form.^58^ The major difference between both types of assays is that ABTS radicals tend to favor reaction through the SPLET mechanism (Sequential Proton Loss Electron Transfer) when in aqueous solutions, while the DPPH radicals react through the SPLET mechanism as well but in solvents like ethanol and methanol.^59−61^ ABTS and DPPH assays were applied to determine the antioxidant effects of the biofabricated green NPs and their corresponding extracts. Data are expressed as radical scavenging % (Figure 8a,b) and as IC_50_ values, which refer to the concentration that causes 50% scavenging of the radicals by the tested sample (Table 5). The results revealed that all the tested samples unveiled considerable antioxidant activity through the scavenging of DPPH and ABTS free radicals in a dose-dependent manner. From the output data of DPPH assay, HQ displayed the highest antioxidant effect against DPPH radicals at all the tested doses with an IC_50_ of 28.6 μg/mL and radical scavenging percentile reaching 91.9% at 100 μg/mL, followed by HC (IC_50_ = 38.8 μg/mL) and HZnC (IC_50_ = 46.02 μg/mL) compared to Trolox reference drug, which demonstrated an IC_50_ < 10 μg/mL. In ABTS assay, HC exhibited the strongest antioxidant effect against ABTS radicals at all the tested doses with an IC_50_ of 21.86 μg/mL and radical scavenging percentile reaching 100% at 100 μg/mL, followed by HQ (IC_50_ = 30.91 μg/mL) and HZnC (IC_50_ = 55.43 μg/mL) compared to Trolox reference drug, which demonstrated an IC_50_ of 3.88 μg/mL. Thus, it can be concluded from the findings that the alcoholic extract-based NPs (HZnC and HFeC) displayed higher radical scavenging effect against DPPH radicals than the aqueous-based ones, while the green Zn NPs revealed higher radical scavenging capacity against ABTS radicals than the green Fe NPs, with HC, HQ, and HZnC as the strongest candidates against both tested radicals.

**Table 5:** IC_50_ Values of the Samples under Study via DPPH and ABTS Assays

	IC_50_ (μg/mL)						
	HC	HQ	HZnC	HFeC	HZnQ	HFeQ	TROLOX
DPPH	38.80	28.60	46.02	102.41	179.95	506.17	<10
ABTS	21.86	30.91	55.43	78.97	77.58	150.25	3.88

Our findings demonstrate a clear link between the flavonoid and phenolic contents of the samples and their ability to scavenge free radicals, as the NPs synthesized using alcoholic extracts exhibit higher TPC, TFC, and superior DPPH and ABTS radical scavenging powers compared to their aqueous-based analogues. This suggests that the phenolic and flavonoid compounds in the alcoholic extracts play a more effective role as capping and coating agents during the synthesis of NPs, thereby enhancing their antioxidant properties against ABTS and DPPH free radicals. Phenolics and flavonoids are well-recognized as the main potent antioxidant phytochemical class.^53^ These observations are consistent with previous studies.^18,19,62^ For example, silver NPs were successfully synthesized using *Satureja intermedia*, which displayed high phenolic and flavonoid content, leading to potent antioxidant activity.^63^ Moreover, these conclusions are supported by our QTOF-LC/MS-MS results, which showed that around 45% of the identified compounds in the extracts belong to phenolic phytochemical classes. Sixty of these phenolics were detected in HC, while 55 were identified in HQ.

### 3.6 Investigation of Skin Anticancer Activity (*In Vitro*)

**Figure 9:**
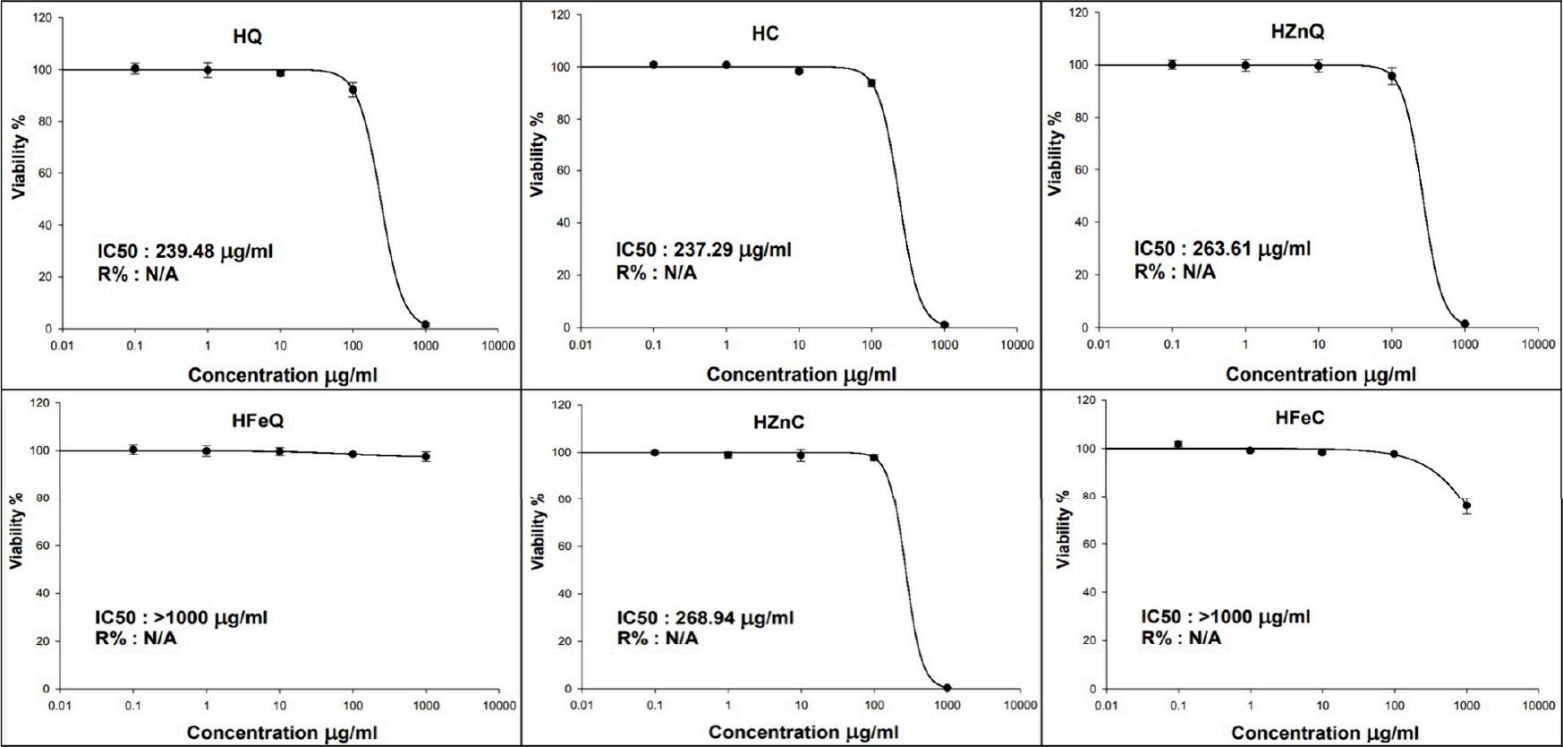
Anticancer activity of LI extracts and their corresponding Fe and Zn NPs against A-431 Human Epidermoid Skin carcinoma. Values are expressed as mean ± SD of three independent experiments.

**Figure 10:**
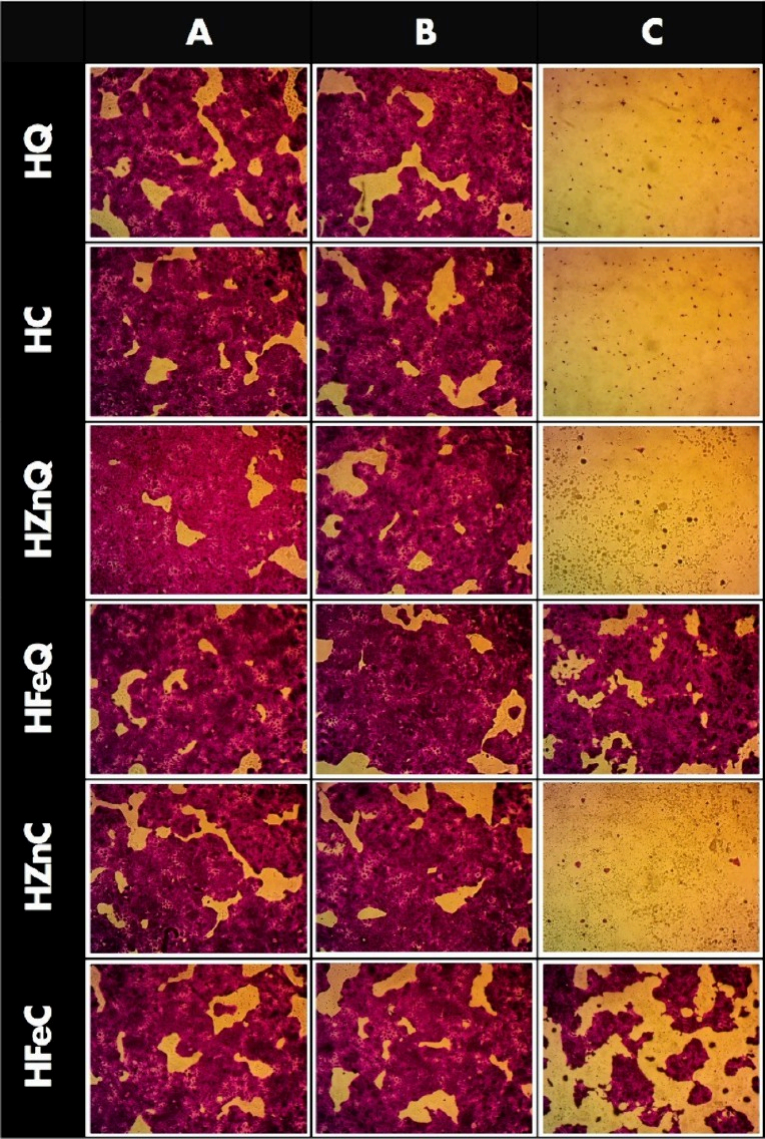
Morphological alterations in control untreated A-431 cell lines and treated cells after 72 h of treatment with LI extracts and their mediated Fe and Zn NPs, (A) 0.1 μg/mL, (B) 10 μg/mL, (C) 1000 μg/mL.

The phyto-fabricated NPs and their corresponding crude extracts were investigated for their anticancer capacity against A-431 human epidermoid skin carcinoma via SRB screening assay. SRB assay is a commonly used method to determine cytotoxicity in cell-based studies through determination of cell density by measuring the cellular protein content. This is because SRB is a xanthene dye that has the ability to attach stoichiometrically to the basic amino acid residues (proteins) in slightly acidic conditions, and it can be detached in basic treatment conditions. Thus, measurement of the quantity of the bound SRB to the cells through colorimetric means can reflect cell density and so cell proliferation.^38^ The results are presented as cell viability % of cancer cells and IC_50_ as shown in Figure 9. Additionally, Figure 10 shows the morphological alterations in the cancer cells upon exposure to the tested samples at different concentrations. It is obvious from the resulted data that the LI-based Zn NPs along with their parent LI alcoholic and aqueous crude extracts are active against the tested cell lines showing a significant decrease of cancer cell viabilities after 72 h exposure, reaching between 0.515% to 1.71% at 1000 μg/mL and IC_50_ ranging between 237.29–268.94 μg/mL. This has been confirmed via microscopical examination of the treated cells as it showed major changes in the cancerous cell morphology like cell detachment and shrinkage, especially at higher concentrations (Figure 10). The alcoholic crude extract demonstrated the strongest effect (IC_50_ = 237.29 μg/mL) among the tested samples, followed by HQ (IC_50_ = 239.48 μg/mL), then their corresponding Zn NPs. In contrast, the LI-based Fe NPs displayed inactivity against the A-431 cell lines. Recently, similar studies were conducted on other plant extracts. For instance, the aerial parts of *Calotropis procera* and *Leptadenia pyrotechnica* were utilized to synthesize iron and zinc NPs, which displayed promising anticancer effects against A-431 human epidermoid skin carcinoma with IC_50_ reaching 188.97 μg/mL. In this study the authors linked the therapeutic capacity of their NPs to its core Fe and Zn metals, beside their phenolic coating content.^18,19^

Thus, herein, the potency of our LI-mediated Zn nanoparticles is believed to stem from the synergistic interaction between the metal elements, which form the core of the NPs, and the phytochemicals from LI, particularly the phenolic compounds coating the nanoparticles. This is supported by various studies highlighting the anticancer properties of Zn nanoparticles, phenolics, and LI, due to their ability to target cancer cells and offer a multimodal approach to cancer treatment.^64−66^ It is hypothesized that the cytotoxicity and genotoxicity of Zn NPs against cancer cells stem from the intracellular release of soluble Zn ions. This release is thought to be facilitated by the acidic conditions prevalent in the tumor microenvironment. This initiates several pathways, leading to cytotoxic effects. The primary mechanism involves zinc-mediated disruption of protein activity, which has severe consequences for cancer cells, including interference with DNA replication and repair, induction of oxidative stress, apoptosis, disruption of the electron transport chain, disturbance of cellular homeostasis, and increased membrane permeability.^65,67^ The second pathway involves a high concentration of released zinc ions, which leads to the generation of reactive oxygen species (ROS). This ROS production is further amplified by the cells’ proinflammatory response to the nanoparticles, as well as the unique surface properties of ZnO nanoparticles, which function as a redox system, continuously generating ROS, which contributes to the nanoparticles’ cytotoxic effects on cancer cells.^68−70^ This is because ROS are potent oxidizing agents, and their accumulation within the cell disrupts the balance of the cell’s oxidative–reductive homeostasis, leading to oxidative stress. This stress results in harmful effects on cellular components, including protein denaturation, lipid peroxidation, and damage to nucleic acids, ultimately causing DNA damage, and triggering cell death through necrosis and apoptosis.^71−74^ Also, many studies reported the potency of henna extracts and phytochemicals as anticancer candidates against various types of cancer cells, especially skin cancer.^66^ Our study results of LC/MS/MS demonstrated the richness of the tested extracts with phenolics, terpenoids, and amino acids. Phenolic compounds are widely recognized for their anticancer properties, acting through multiple mechanisms. These mechanisms include induction of apoptosis in cancer cells, besides promoting differentiation and regulating processes like inflammation, angiogenesis, and metastasis. These effects are achieved, in part, by modulating the redox balance of cells under oxidative stress.^75,76^ For instance, several phenolic compounds, including quercetin, ellagic acid, apigenin, and resveratrol, have been reported to inhibit carcinogenesis by inducing apoptosis through two primary pathways: extrinsic and intrinsic. In the extrinsic pathway, apoptosis is initiated by the activation of cell surface receptors, such as tumor necrosis factor-α (TNF-α), which subsequently triggers caspase-8 activation. In the intrinsic pathway, apoptosis is regulated by internal cell signaling through the mitochondria. This process is triggered by various mitochondrial proteins, including inhibitors of small mitochondrial-derived activators of caspases (SMACs), apoptosis proteins (IAPs), and B-cell lymphoma-2 protein (Bcl-2). These proteins, along with mitochondrial membrane integrity and polarity, play key roles in the initiation and regulation of this pathway.^75,77^ Our metabolic profiling results also indicated the presence of different types of terpenoids, which have been demonstrated to have potency as anticancer agents, especially for skin cancer melanoma. Cytotoxic effect of terpenoids takes place via diverse pathways and mechanisms, including induction of cell cycle arrest, angiogenesis suppression, decreased tumor cell differentiation, proliferation, and metastasis, as well as apoptosis.^78,79^ Furthermore, amino acids are pivotal in addressing cancer cells, as certain metabolic intermediates derived from amino acids contribute simultaneously to both tumorigenic and antitumorigenic processes. For example, nitric oxide (NO), a byproduct of arginine metabolism via the citrulline–NO pathway, can facilitate tumor growth by promoting angiogenesis. Conversely, it can function as a tumor suppressor by upregulating the p53 protein.^80^ Beside the NPs’ composition, the small size of the fabricated NPs could be an extra synergistic factor that contributes to the therapeutic potential of the formed NPs since their tiny sizes enhance their cellular uptake, bioavailability, and distribution within the cells, making them outstanding candidates for targeted drug delivery.^3−7^

## 4 Conclusion

This work revealed the effectiveness of both aqueous and 80% ethanolic extracts of LI as reducing, capping, coating, and stabilizing agents in the green synthesis of Fe and Zn NPs for potential biomedical applications. Notably, the alcoholic extract of LI demonstrated superior surface coating capabilities for the biosynthesized Zn and Fe NPs compared to the aqueous extract, as shown by SEM and EDX analysis, which revealed a lower propensity for agglomeration and a higher carbon content compared with NPs synthesized using the aqueous extract. In contrast, the higher concentrations of Zn and Fe in the aqueous extract-derived NPs, compared to those synthesized with alcoholic extracts, suggest that the aqueous extract had greater efficacy as a reducing agent during the biosynthesis of Zn and Fe NPs. The TPC and TFC further supported these observations, indicating a higher phenolic and flavonoid content in the alcoholic extract-based NPs. Furthermore, coating the NPs with phytochemicals from the extracts was confirmed via FT-IR spectroscopy, showing the characteristic bands of functional groups. Additionally, variations in bands’ intensities or their absences in the NPs, relative to their corresponding extracts, confirmed the participation of these phytochemical constituents in the reduction process during NP synthesis. These findings were supported by our QTOF-LC/MS-MS and multivariate analyses, which revealed that approximately 45% of the detected compounds in the extracts belong to the phenolic phytochemical classes. Specifically, 60 phenolic compounds were determined in the HC extract, while 55 were pinpointed in the HQ extract. In addition, the OPLS-DA modeling demonstrated that 52.5% of the assigned marker compounds of HC and 46.6% of the markers of HQ belong to phenolics. Due to this effective coating, the Zn NPs synthesized with the alcoholic extract (HZnC) exhibited the strongest antioxidant activity against DPPH and ABTS radicals among the tested NPs. All the tested samples, except the Fe NPs, demonstrated promising cytotoxic effect against A-431 human epidermoid carcinoma cells, with HC and HZnQ as the strongest among the investigated extracts and NPs, respectively, reaching cell viability up to 1% at 1000 μg/mL after 72 h of exposure. This study emphasized that the extract type used in the synthesis process affects the physicochemical and biological behavior of its corresponding NPs. Therefore, it is recommended to decide on the type of the used extract based on in-depth chemical and biological studies of the parent possible extracts of the plant to get the optimum NPs for your sought application. Beside this, the findings suggest that the green-synthesized NPs have potential as sustainable anticancer and antioxidant agents and may hold promise for other biomedical applications. Therefore, we recommend further chemical and biological studies to elucidate their mechanisms of action, in vivo bioavailability, effectiveness, and kinetics. This study appears to be the first to explore the biological synthesis of green Zn and Fe nanoparticles using two diverse types of LI extracts, addressing the research gap regarding the most suitable extraction protocol for green NPs’ fabrication, particularly for biomedical applications.

## Data Availability

All data generated and analyzed during this study are included in this published article.

## Supporting Information Available

Raw data figures for XRD patterns, UV–vis spectra, and QTOF-LC/MS/MS Chromatograms (PDF)
