# Supplementary material for: Alchemy in Nature: The Role of Lawsonia inermis Extract Choice in Crafting Potent Anticancer Metal Nanoparticles
Source: ACS Appl Mater Interfaces. 2025 Jan 11;17(3):4637–61. doi: 10.1021/acsami.4c19585 (PMC11759054; doi:10.1021/acsami.4c19585)
Supplement: Supplementary file 1 — am4c19585_si_001.pdf [file am4c19585_si_001.pdf]

<u>**Supporting Information**</u>

# Title: Alchemy in Nature: The Role of *Lawsonia inermis* Extract Choice in Crafting Potent Anticancer Metal Nanoparticles

Rana Ahmed El-Fitiany [a,b] , Riham El Nahas [a], Seba Al Balkhi [a], Shouq Aljaeedi [a], Afra Alblooshi [a], Fathy M. Hassan [a], Abbas Khaleel [a], Abdelouahid Samadi [a], Mohammad A. Khasawneh [a*]

[a] Department of Chemistry, College of Science, United Arab Emirates University, Al Ain, P.O. Box No. 15551, United Arab Emirates

[b] Pharmacognosy Department, Faculty of Pharmacy, Egyptian Chinese University, Cairo, P.O. Box No. 11734, Egypt

**<u>*Corresponding author's information:</u>**

**Name:** Mohammad Ahmad Khasawneh.

**Full address:** United Arab Emirates University, Sheikh Khalifa Bin Zayed St, Asharij, Al-Ain, Abu Dhabi

**Cellular phone number:** +971 55 937 6770

**Email address:** mohammad.khasawneh@uaeu.ac.ae

**Email addresses of all authors:**

| Name | E-mail | Phone number | ORCID iD |
|---|---|---|---|
| Rana Ahmed El-Fitiany | 202190078@uaeu.ac.ae | +971501853867 | 0000-0002-4477-1873 |
| Riham El Nahas | 201950263@uaeu.ac.ae | +971557801914 | |
| Seba Al Balkhi | 201950256@uaeu.ac.ae | +971508331621 | |
| Shouq Aljaeedi | 201709079@uaeu.ac.ae | +971507019992 | |
| Afra AlBlooshi | aalblooshi@uaeu.ac.ae | +971509944751 | 0000-0002-8565-0111 |
| Fathy M. Hassan | f.hassan@uaeu.ac.ae | +971503367131 | 0000-0002-4884-7820 |
| Abbas Khaleel | abbask@uaeu.ac.ae | +971506937292 | 0000-0002-0346-384X |
| Abdelouahid Samadi | samadi@uaeu.ac.ae | +971568049690 | 0000-0003-1766-4471 |
| Mohammad A. Khasawneh | mohammad.khasawneh@uaeu.ac.ae | +971559376770 | 0000-0001-5853-8311 |


**Running title: Metal Nanoparticles of *Lawsonia inermis***

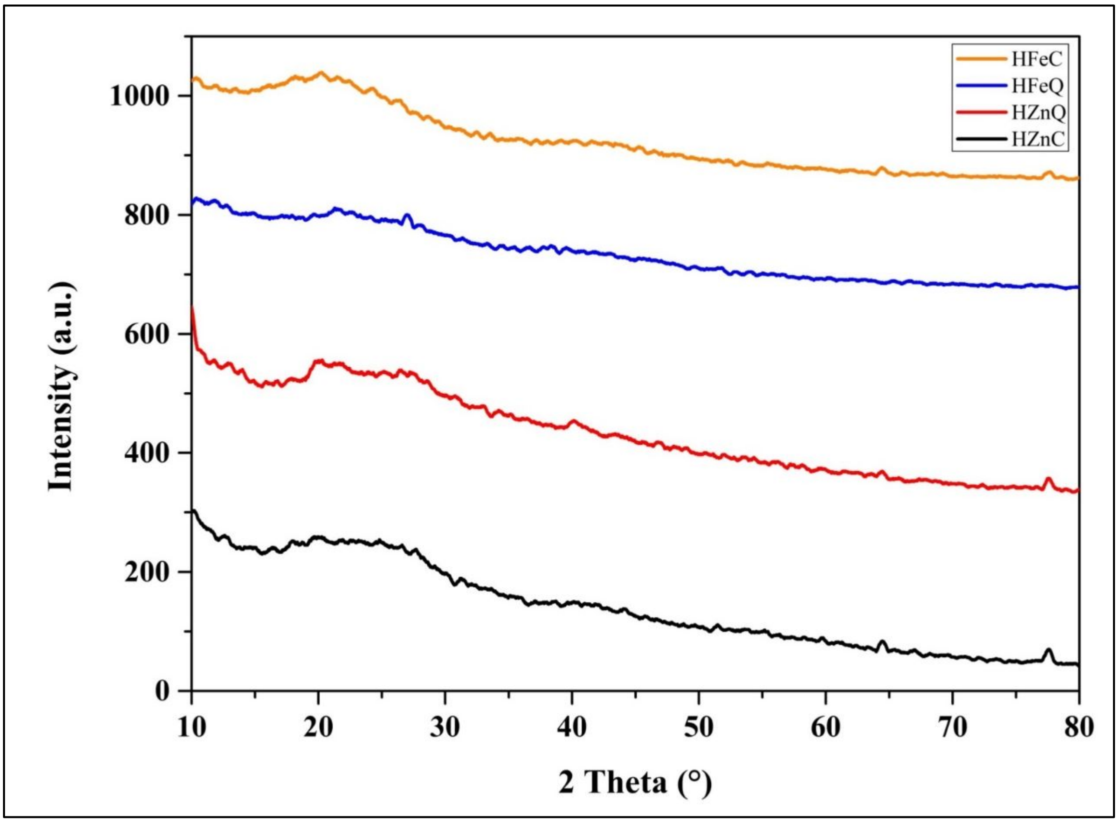


**Figure S1: XRD patterns of LI-based Zn and Fe NPs**

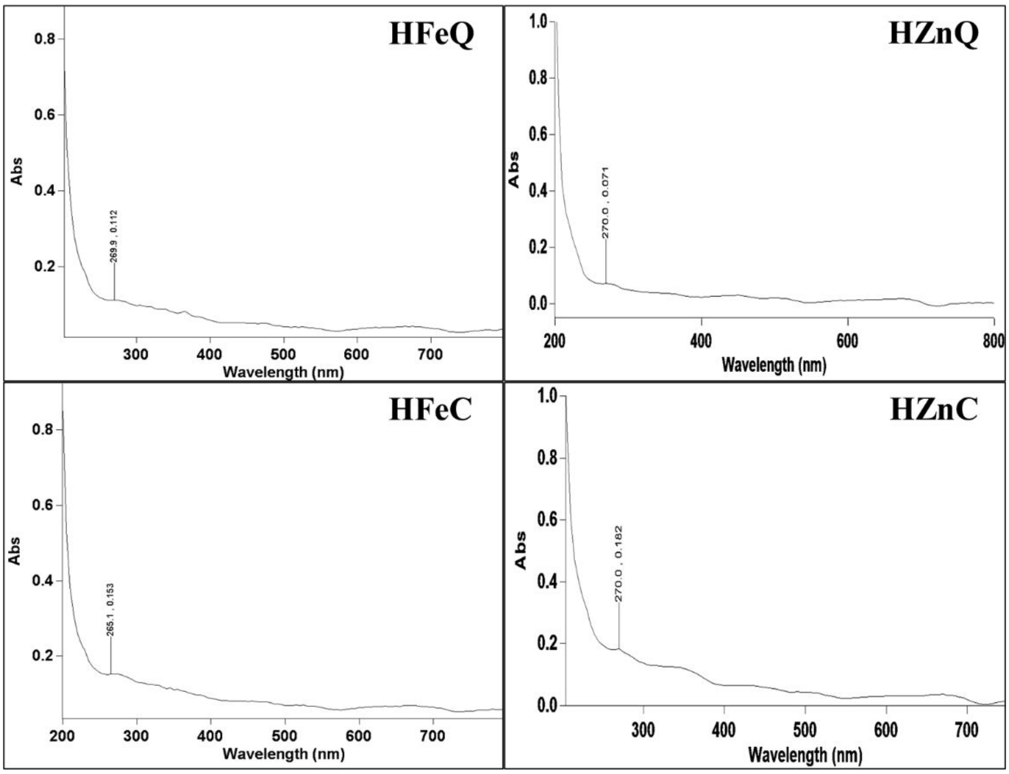


**Figure S2: UV-Vis spectra of the fabricated green NPs**

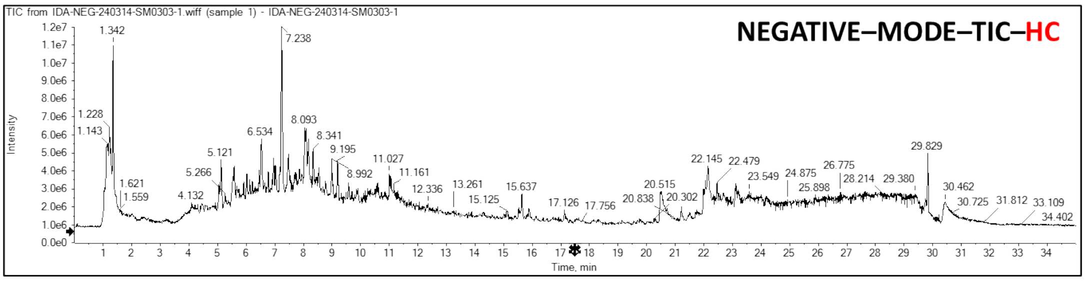


**Figure S3: Total intensity chromatogram (TIC) of the ethanolic extract (HC) of LI in the negative mode**

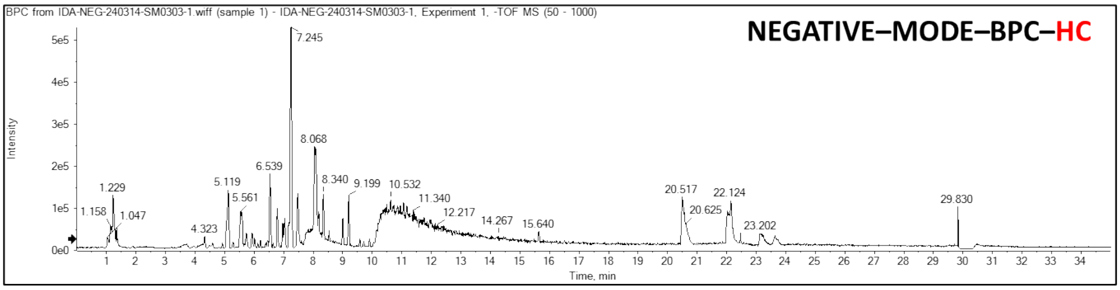


**Figure S4: Base peak chromatogram (BPC) of the ethanolic extract (HC) of LI in the negative mode**

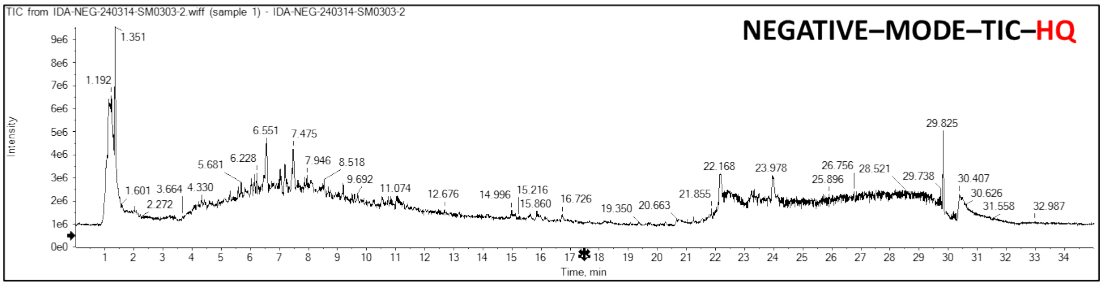


**Figure S5: Total intensity chromatogram (TIC) of the aqueous extract (HQ) of LI in the negative mode**

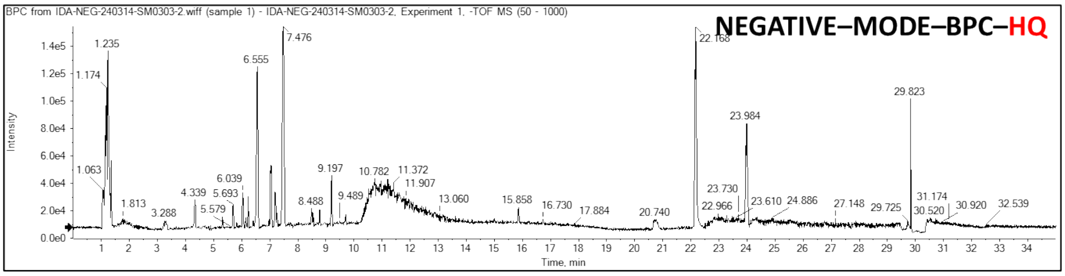


**Figure S6: Base peak chromatogram (BPC) of the aqueous extract (HQ) of LI in the negative mode**

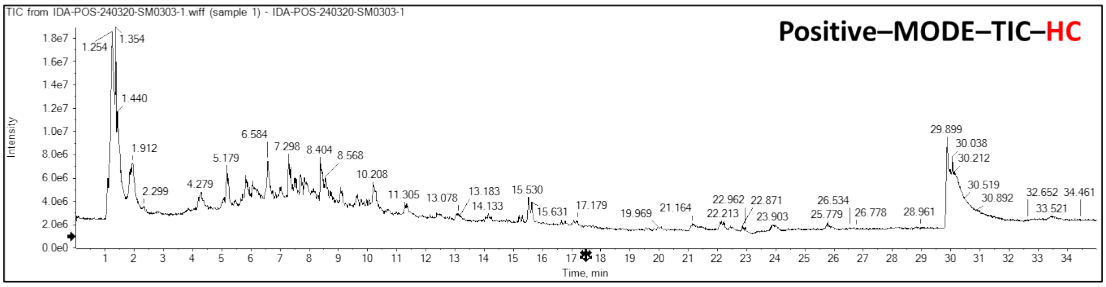


**Figure S7: Total intensity chromatogram (TIC) of the ethanolic extract (HC) of LI in the positive mode**

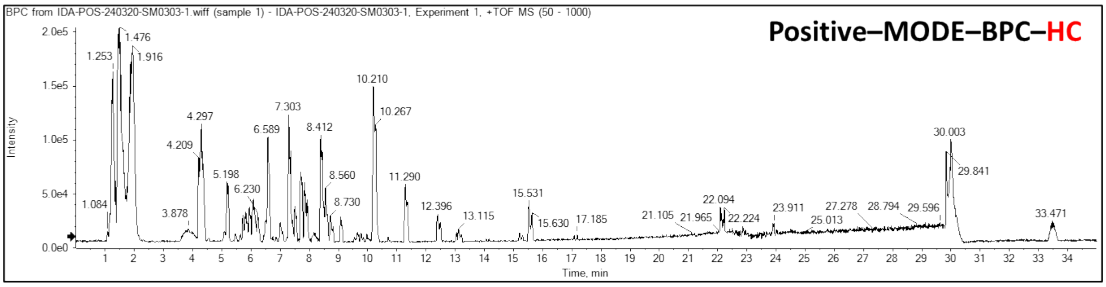


**Figure S8: Base peak chromatogram (BPC) of the ethanolic extract (HC) of LI in the positive mode**

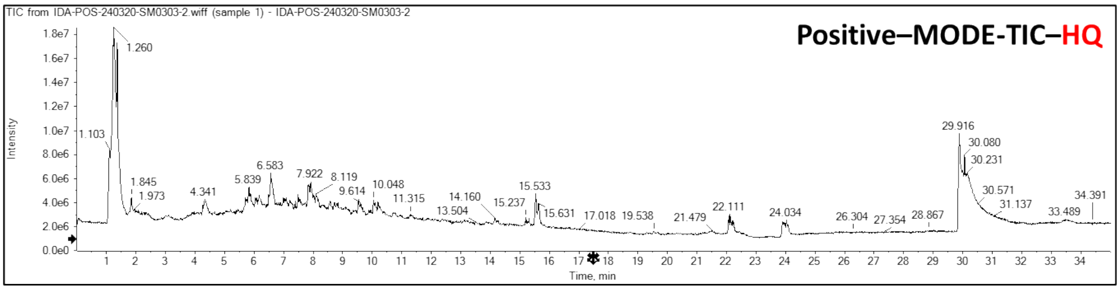


**Figure S9: Total intensity chromatogram (TIC) of the aqueous extract (HQ) of LI in the positive mode**

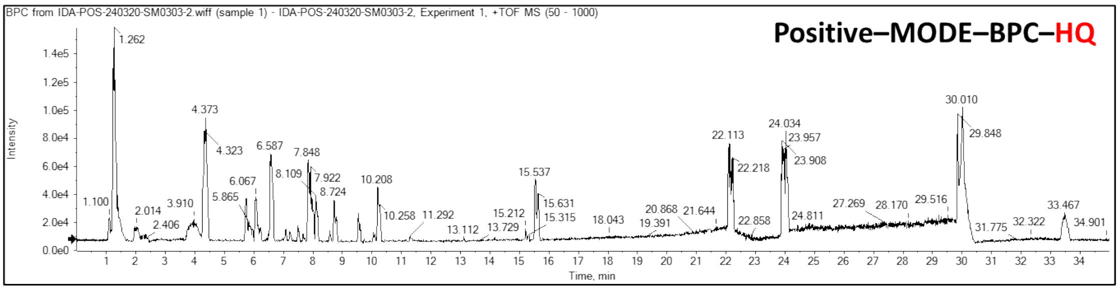


**Figure S10: Base peak chromatogram (BPC) of the aqueous extract (HQ) of LI in the positive mode**
